# The *Mir181ab1* cluster promotes *KRAS*-driven oncogenesis and progression in lung and pancreas

**DOI:** 10.1172/JCI129012

**Published:** 2020-03-09

**Authors:** Karmele Valencia, Oihane Erice, Kaja Kostyrko, Simone Hausmann, Elizabeth Guruceaga, Anuradha Tathireddy, Natasha M. Flores, Leanne C. Sayles, Alex G. Lee, Rita Fragoso, Tian-Qiang Sun, Adrian Vallejo, Marta Roman, Rodrigo Entrialgo-Cadierno, Itziar Migueliz, Nerea Razquin, Puri Fortes, Fernando Lecanda, Jun Lu, Mariano Ponz-Sarvise, Chang-Zheng Chen, Pawel K. Mazur, E. Alejandro Sweet-Cordero, Silvestre Vicent

**Affiliations:** 1 University of Navarra, Center for Applied Medical Research, Program in Solid Tumors, Pamplona, Spain.; 2 University of Navarra, Department of Biochemistry and Genetics, Pamplona, Spain.; 3 Centro de Investigación Biomédica en Red de Cáncer (CIBERONC), Madrid, Spain.; 4 Division of Hematology and Oncology, UCSF, San Francisco, California, USA.; 5 Department of Experimental Radiation Oncology, University of Texas MD Anderson Cancer Center, Houston, Texas, USA.; 6 Bioinformatics Platform, Center for Applied Medical Research, Pamplona, Spain.; 7 IdiSNA, Navarra Institute for Health Research, Pamplona, Spain.; 8 Department of Pediatrics, and; 9 Department of Microbiology and Immunology, Stanford University School of Medicine, Stanford, California, USA.; 10 Achelois Oncology, Redwood City, California, USA.; 11 University of Navarra, Department of Pathology, Anatomy and Physiology, Pamplona, Spain.; 12 University of Navarra, Center for Applied Medical Research, Program in Gene Therapy and Regulation of Gene Expression, Pamplona, Spain.; 13 Genetics Department, Yale University, New Haven, Connecticut, USA.; 14 Clínica Universidad de Navarra, Department of Medical Oncology, Pamplona, Spain.

**Keywords:** Oncology, Mouse models, Noncoding RNAs, Oncogenes

## Abstract

Few therapies are currently available for patients with *KRAS*-driven cancers, highlighting the need to identify new molecular targets that modulate central downstream effector pathways. Here we found that the microRNA (miRNA) cluster including miR181ab1 is a key modulator of *KRAS*-driven oncogenesis. Ablation of *Mir181ab1* in genetically engineered mouse models of *Kras*-driven lung and pancreatic cancer was deleterious to tumor initiation and progression. Expression of both resident miRNAs in the *Mir181ab1* cluster, miR181a1 and miR181b1, was necessary to rescue the *Mir181ab1*-loss phenotype, underscoring their nonredundant role. In human cancer cells, depletion of miR181ab1 impaired proliferation and 3D growth, whereas overexpression provided a proliferative advantage. Lastly, we unveiled miR181ab1-regulated genes responsible for this phenotype. These studies identified what we believe to be a previously unknown role for miR181ab1 as a potential therapeutic target in 2 highly aggressive and difficult to treat *KRAS*-mutated cancers.

## Graphical abstract

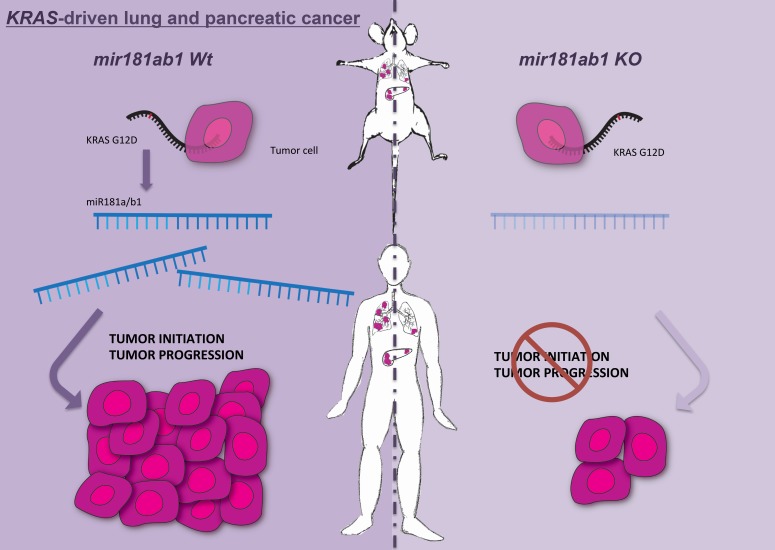

## Introduction

*KRAS* is one of the most commonly mutated oncogenes in human cancer (1) and is a key oncogenic driver in many lung and most pancreatic cancers (2–5). KRAS induces the coordinated action of several downstream effector pathways to induce a transcriptional response that sustains a pro-oncogenic phenotype (1). Prior work has identified genes that are transcriptionally regulated as a consequence of KRAS activation (6–10), fostering strategies for the discovery of critical transcriptional regulators within the KRAS signaling pathway (11, 12). An alternative mechanism for regulation of gene expression is via microRNAs (miRNAs) (13). However, our understanding of the role of miRNAs functionally regulating the consequences of KRAS activation is still limited (14–17). The study of miRNA function is an alternative strategy to yield molecular insights necessary for the development of novel therapies against KRAS-driven tumors.

miRNAs are small, noncoding RNAs that act largely by fine-tuning posttranscriptional gene expression (13). Many miRNAs are differentially expressed in human tumors (18–22) and a few have been confirmed to have either oncogenic or tumor-suppressive effects across tumor types (23–25). Some miRNAs are themselves regulated by oncogenic pathways. For example, pioneering studies described overexpression of MYC (26) or mutations in *TP53* (27–31) as drivers of miRNA expression. Comparatively fewer studies have focused on miRNAs with a pro-oncogenic role in the context of mutant-*KRAS* tumorigenesis (14, 15, 32, 33), in contrast with the wealth of information about tumor-suppressive miRNAs reported to downregulate KRAS expression (34, 35). In addition, functional validation of miRNAs involved in KRAS-driven oncogenesis has focused primarily on the role of such miRNAs in tumor initiation (14, 15), with less attention on their role in tumor progression. To our knowledge, no miRNAs clearly functioning in tumor maintenance in KRAS-driven cancers have definitively been identified. Understanding the role of miRNAs sustaining *KRAS* oncogene tumorigenesis might unveil new targets amenable to intervention strategies.

We identified *MiR181ab1* as a miRNA cluster upregulated by oncogenic KRAS and used multiple genetically engineered mouse models (GEMMs) to demonstrate a role for this cluster in both initiation and maintenance of lung and pancreatic cancer. We extended these results to human cells where we show that miR181ab1 plays an important role in early and late stages of KRAS-driven oncogenesis. Our findings support the value of mouse genetics for the identification of functionally relevant elements of the KRAS signaling network and justify further efforts to develop inhibitory strategies against members of the *MiR181ab1* cluster as a possible therapeutic opportunity in *KRAS*-mutated tumors.

## Results

### Deletion of Mir181ab1 impairs Kras-driven lung cancer development.

We used mouse embryonic fibroblasts (MEFs) carrying a conditionally activatable allele to identify miRNAs upregulated by oncogenic KRAS (36). Differentially expressed miRNAs were profiled using a bead-based flow cytometric method (37). Fifty-three upregulated and 5 downregulated miRNAs were identified in MEFs expressing oncogenic KRAS compared with controls (log fold change [logFC] >1 or < 1) (Supplemental Figure 1A; supplemental material available online with this article; https://doi.org/10.1172/JCI129012DS1). The miR181 family (miR181a, miR181b, miR181c, and miR181d) were among the top upregulated miRNAs (Supplemental Figure 1, A and B). Thus, we focused on this miRNA family for subsequent experiments.

**Figure 1:**
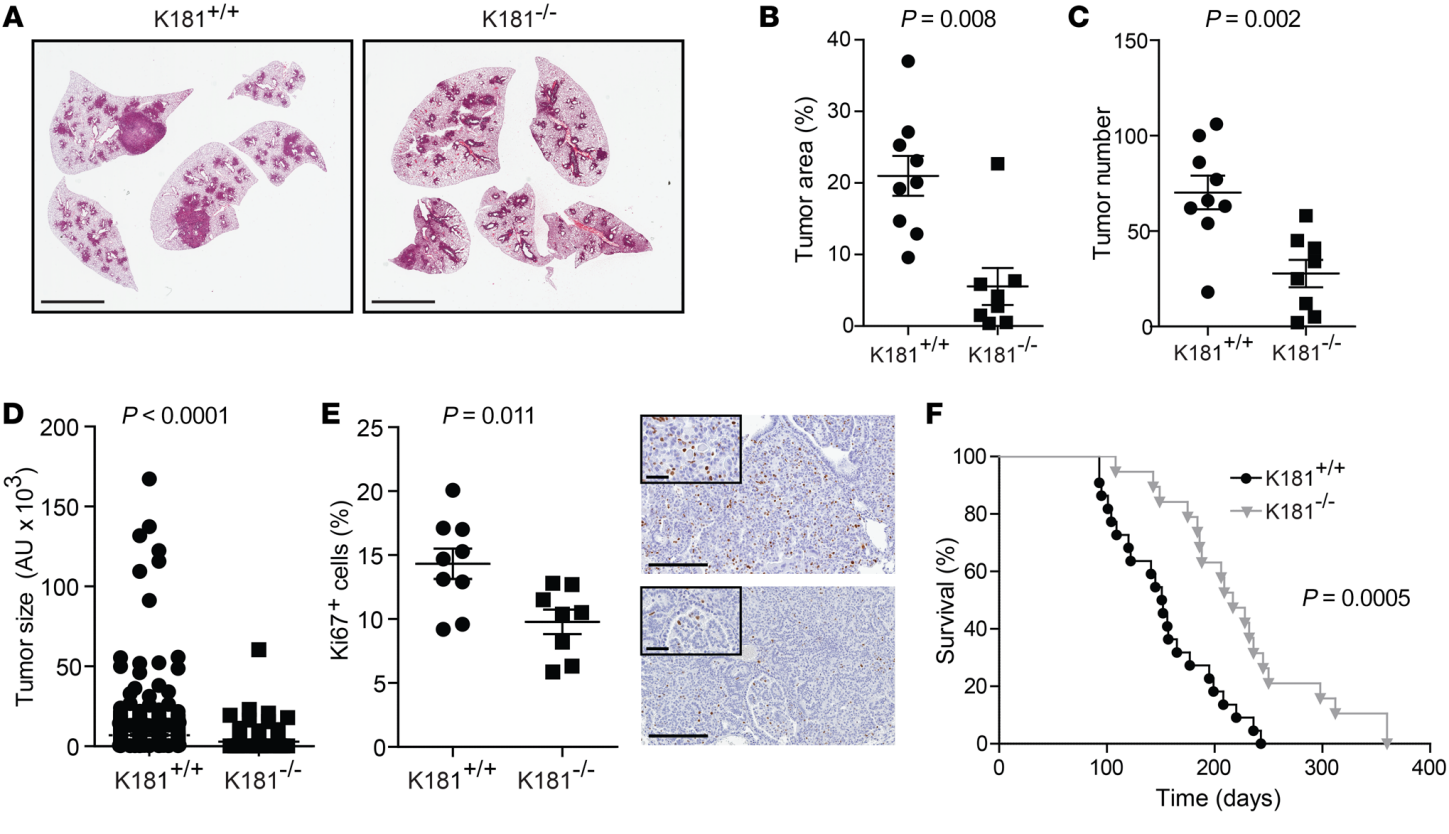
Systemic *Mir181ab1* ablation impairs *Kras*-driven lung cancer. (**A**) Representative H&E-stained sections of K181^+/+^ and K181^–/–^ lungs 20 weeks after adCre infection. Scale bars: 5 mm. (**B**) Average tumor area percentage in K181^+/+^ (*n* = 9) and K181^–/–^ (*n* = 8) groups compared by *t* test. (**C**) Mean number of tumors per mouse in K181^+/+^ (*n* = 9) and K181^–/–^ (*n* = 8) mice compared by *t* test. (**D**) Average tumor size in K181^+/+^ (*n* = 632) and K181^–/–^ (*n* = 222) groups compared by Mann-Whitney *U* test. (**E**) Left: Average percentage of Ki67^+^ cells in tumors from K181^+/+^ (*n* = 9) and K181^–/–^ (*n* = 8) mice compared by *t* test. Right: Immunohistochemistry for Ki67 expression in representative sections. Scale bars: 200 μm and 50 μm (insets). (**F**) Kaplan-Meier plot of K181^+/+^ (*n* = 23, median survival = 151.5 days) and K181^–/–^ (*n* = 19, median survival = 217 days) mice (log-rank test).

To evaluate the role of miR181ab1 in tumor initiation, *Mir181ab1*^–/–^ mice (38) were crossed to *Kras*^LSL-G12D/+^ mice to generate *Kras*^LSL-G12D/+^ *Mir181ab1*^–/–^. Compound mutant *Kras*^LSL-G12D/+^ *Mir181ab1*^+/+^ (K181^+/+^) and *Kras*^LSL-G12D/+^ *Mir181ab1*^–/–^ (K181^–/–^) mice were treated with intranasal instillation of adenovirus containing Cre recombinase (adCre) to evaluate the function of *Mir181ab1* in KRAS-driven oncogenesis. Histological analysis of hematoxylin and eosin–stained (H&E-stained) sections of mouse lungs 20 weeks after adCre revealed that *Mir181ab1* deletion significantly reduced overall tumor burden (Figure 1, A and B), with both tumor number and tumor size decreased in K181^–/–^ mice (Figure 1, C and D). The effect on both tumor number and size suggested an effect on both tumor initiation and progression, possibly due to impaired proliferative capacity as indicated by fewer Ki67^+^ cells (Figure 1E). Analysis of individual tumors by laser microdissection showed a substantial reduction in both miR181a and miR181b in K181^–/–^ mice, with no compensatory increase in miR181c or miR181d (Supplemental Figure 1, C and D). As miR181 can be expressed from 3 different clusters in mouse chromosomes 1, 2, and 8 (chromosomes 1, 9, 19 in human) (Supplemental Figure 1E), these data suggest that neither the *Mir181ab2* nor the *Mir181cd8* cluster compensates for the loss of miR181ab1 in this model. Furthermore, *Mir181ab1* loss significantly increased overall survival in mice harboring *Kras* mutations (Figure 1F). Taken together, these results demonstrate that the *Mir181ab1* cluster has a prominent role in *Kras*-dependent lung tumorigenesis.

**Figure 2:**
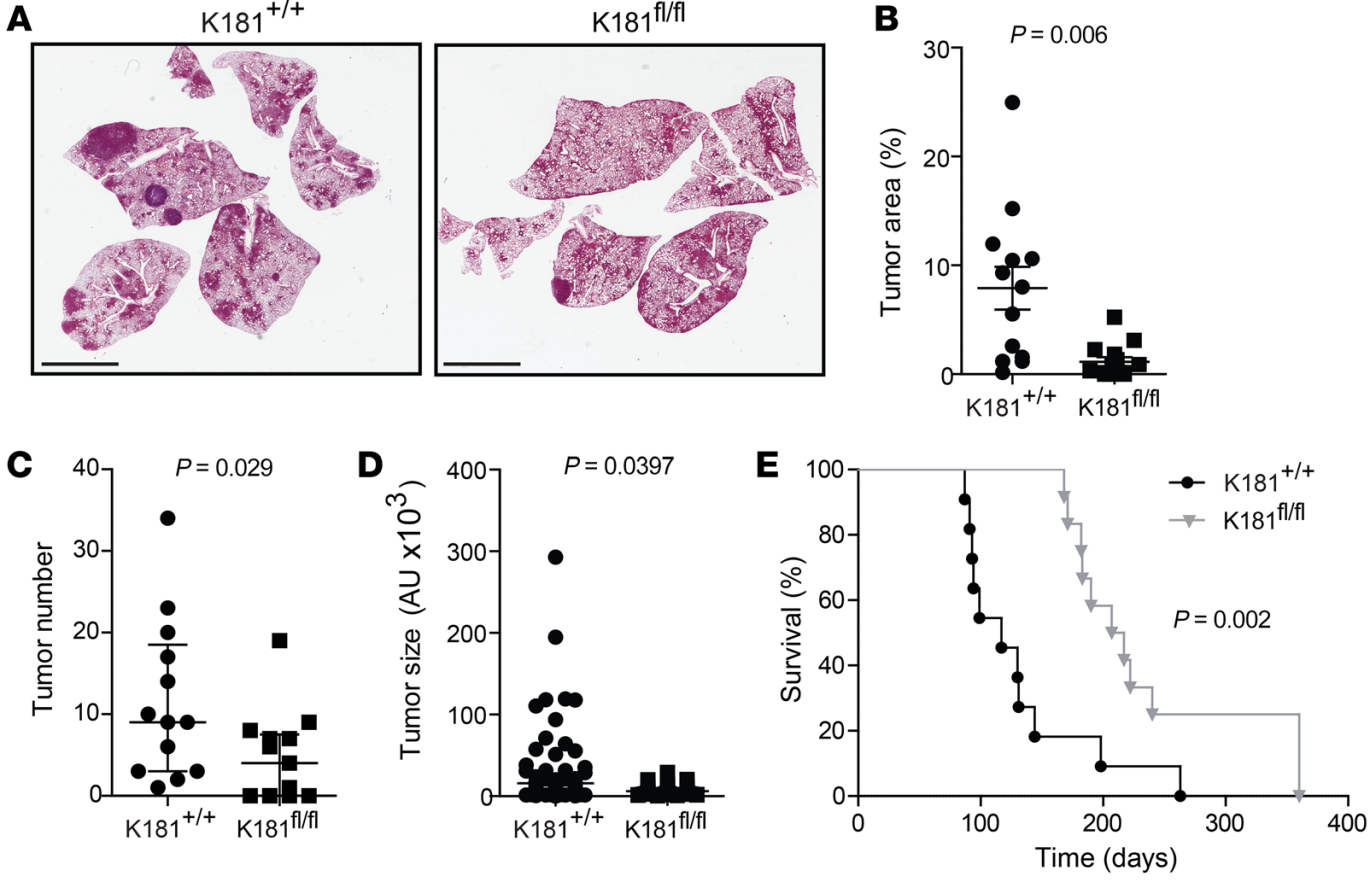
Conditional *Mir181ab1* knockout negatively impacts lung cancer formation. (**A**) Representative H&E-stained sections of K181^+/+^ and K181^fl/fl^ lungs 20 weeks after adCre infection. Scale bars: 5 mm. (**B**) Quantification of tumor area in K181^+/+^ (*n* = 13) and K181^fl/fl^ (*n* = 14) mice compared by *t* test. (**C**) Mean number of tumors per mouse in K181^+/+^ (*n* = 13) and K181^fl/fl^ (*n* = 14) mice compared by Mann-Whitney *U* test. (**D**) Average tumor size of K181^+/+^ (*n* = 151) and K181^fl/fl^ (*n* = 61) mice compared by *t* test. Error bars correspond to SEM. (**E**) Kaplan-Meier plot of K181^+/+^ (*n* = 10, median survival = 117 days) and K181^fl/fl^ (*n* = 13, median survival = 212 days) mice (log-rank test).

Intranasal administration of adCre to *Kras*^LSL-G12D/+^ mice produces an inflammatory response involving the recruitment of T and B cells, and this reaction is essential for the development of lung adenomas (39). Importantly, *Mir181ab1*^–/–^ mice show severe defects in lymphoid development and in T cell homeostasis and function (38, 40–42). Therefore, it is possible that the effect of *Mir181ab1* depletion could be due to modulation of the tumor immune microenvironment. To determine whether the differences in tumor development between K181^+/+^ and K181^–/–^ mice were due to loss of miR181ab1 expression in T cells or other non–cell autonomous effects, we conditionally deleted the *Mir181ab1* cluster in lung epithelial cells using *Mir181ab1^fl/fl^* mice (38). *Kras*^LSL-G12D/+^ mice were bred to *Mir181ab1^fl/fl^* mice to yield compound K181^+/+^ and *Kras*^LSL-G12D/+^ *Mir181ab1^fl/fl^* (K181^fl/fl^) mice. Twenty weeks following adCre, analysis of H&E-stained sections revealed a significant decrease in the K181^fl/fl^ mice compared with the K181^+/+^ group (Figure 2, A and B), similar to that observed in K181^–/–^ mice. A reduction in the average number of tumors and tumor size was also found (Figure 2, C and D). Conditional deletion of *Mir181ab1* in lung epithelial cells also increased mouse survival (Figure 2E). Taken together, these results demonstrate that miR181ab1 expression in lung epithelial tumor cells contributes to formation of *Kras* oncogene–initiated tumors.

### Deletion of Mir181ab1 impairs Kras-driven pancreatic ductal adenocarcinoma tumorigenesis.

**Figure 3:**
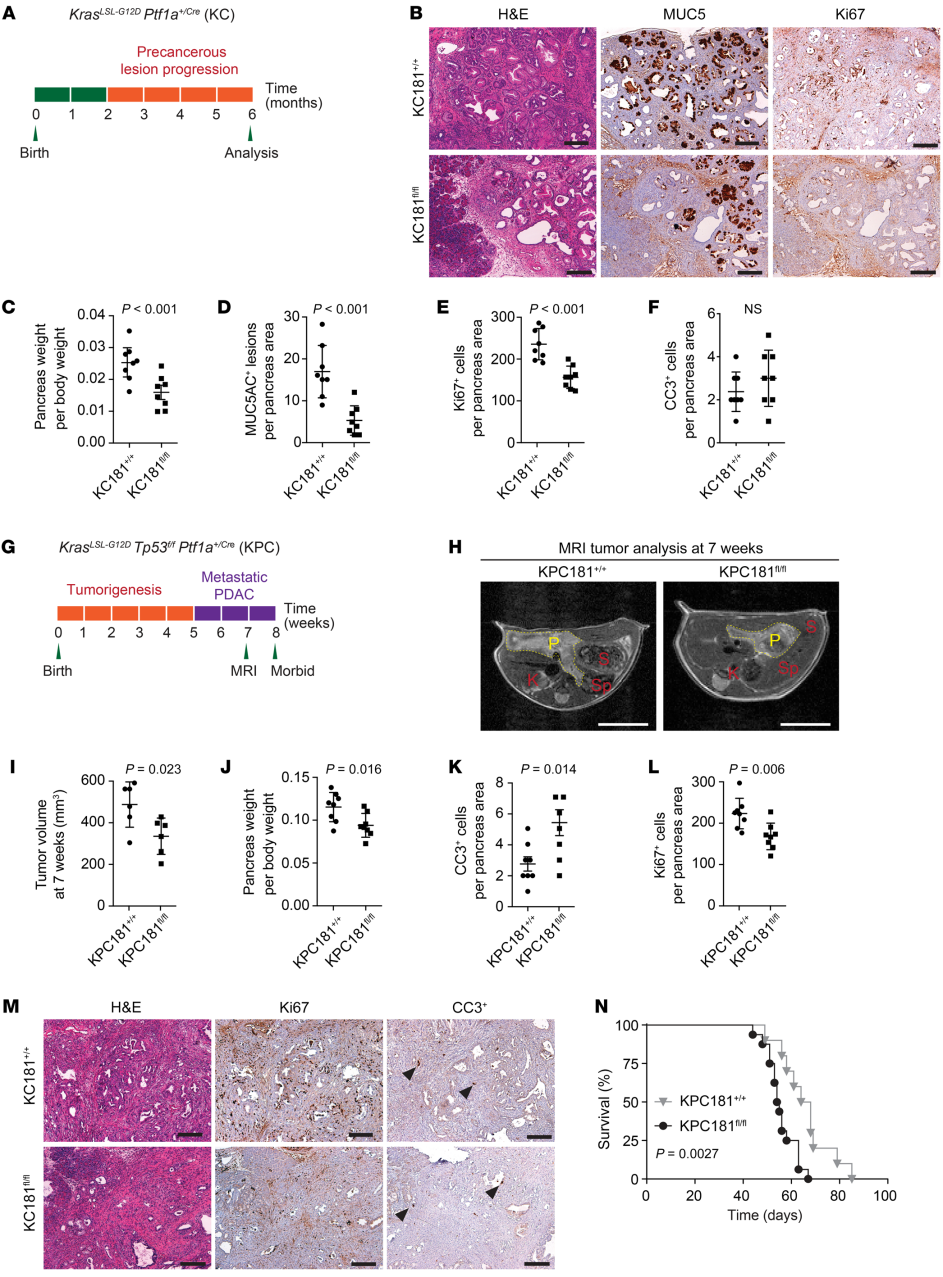
*Mir181ab1* deletion represses *Kras*-driven pancreatic tumorigenesis in vivo. (**A**) Schematic representation of the tumor initiation experiment used to measure precancerous (PanINs) lesion formation. (**B**) Representative H&E-stained sections and IHC for MUC5AC, a marker of PanIN lesions, and Ki67, a marker of cell proliferation (*n* = 8). Scale bars: 100 μm. (**C**) Quantification of pancreas weight at 6 months in KC181^fl/fl^ (*n* = 8) and control KC181^+/+^ (*n* = 8) mice compared by *t* test. (**D**–**F**) Quantification of MUC5AC-positive lesions, Ki67-positive proliferating cells, and cleaved caspase-3–positive (CC3-positive) apoptotic cells in KC181^fl/fl^ (*n* = 8) and control KC181^+/+^ (*n* = 8) mice. Data were compared by *t* test. (**G**) Schematic representation of the tumor progression experiment used to measure PDAC development. (**H**) Representative MRI scan of 7-week-old KPC181^fl/fl^ and KPC181^+/+^ mutant mice. Yellow dotted lines indicate pancreas area. P, pancreas; S, stomach; K, kidney; Sp, spleen. Scale bars: 1 cm. (**I**) Tumor volume quantification in KPC181^fl/fl^ and KPC181^+/+^ mutant mice at 7 weeks of age based on MRI scan (*n* = 6 per group). Data were compared by *t* test and are represented as mean ± SEM. (**J**) Quantification of pancreas weight to body weight in KPC181^fl/fl^ and KPC181^+/+^ (*n* = 8 mice/group) compared by *t* test. (**K** and **L**) Quantification of CC3-positive proliferating cells and Ki67-positive apoptotic cells in pancreatic tumors of KPC181^fl/fl^ and KPC181^+/+^ mutant mice (*n* = 8 mice/group) compared by *t* test. (**M**) Representative H&E and IHC for Ki67 and CC3 in pancreatic tumors of KPC181^fl/fl^ and KPC181^+/+^ mice. Scale bars: 100 μm. (**N**) Kaplan-Meier survival curves for KPC181^+/+^ mice (*n* = 16; median survival = 54.5 days) and KPC181^fl/fl^ mice (*n* = 10; median survival = 66 days) (log-rank test).

To determine if *MiR181ab1* plays a functional role in other oncogenic KRAS–driven cancers, we evaluated its role in pancreatic ductal adenocarcinoma (PDAC), a highly lethal cancer in which *KRAS* mutations are present in over 90% of cases. PDAC shows overexpression of miR181a, miR181b, and miR181c relative to benign pancreatic tissue (43, 44) and expression of miR181b negatively correlates with PDAC patient survival (45). We studied the effect of *MiR181ab1* in early stages of pancreatic tumorigenesis by deleting the cluster and activating expression of oncogenic *Kras*^LSL-G12D/+^ in mouse pancreas using a *Ptf1a*^Cre/+^ strain (46). In the *Ptf1a*^Cre/+^ *Kras*^LSL-G12D/+^ mice, pancreatic intraepithelial neoplasia (PanIN) are observed around 6 months of age (Figure 3A). Cohorts of *Ptf1a*^Cre/+^ *Kras*^LSL-G12D/+^ (KC181^+/+^) and *Ptf1a*^Cre/+^ *Kras*^LSLG12D^ *Mir181ab1*^fl/fl^ (KC181^fl/fl^) mice were obtained. Deletion of *Mir181ab1* greatly reduced tumor weight and decreased PanIN lesions (assessed by MUC5AC) (Figure 3, B–D). Immunohistochemistry (IHC) revealed a decrease in the number of proliferating cells without changes in the number of apoptotic cells in KC181^fl/fl^ mice compared with controls (Figure 3, B, E, and F).

Next, we assessed miR181ab1 function in pancreatic tumor development using the *Ptf1a*^+/Cre^ *Kras*^+/LSL-G12D^ *Trp53*^fl/fl^ (KPC181^fl/fl^) mutant model in which PDAC develops with 100% penetrance 6–8 weeks after birth (Figure 3G) (47). Magnetic resonance imaging (MRI) revealed that tumor volume in *Mir181ab1* knockouts was significantly reduced compared with age-matched control mice (Figure 3, H and I), consistent with a reduced pancreas weight (Figure 3J). At autopsy, pancreatic tissue from control KPC181^+/+^ mutant mice was entirely occupied by transformed cells, whereas in KPC181^fl/fl^ mutant mice areas of normal pancreatic tissue remained with decreased signal for Ki67 and elevated number of cleaved caspase-3–positive (CC3-positive) cells compared with control animals (Figure 3, K–M). Furthermore, *Mir181ab1* ablation prolonged overall survival in this aggressive model (Figure 3N). Taken together, these data support a key in vivo role for *Mir181ab1* in oncogenic *Kras*–driven pancreatic tumorigenesis.

### The Mir181ab1 cluster is required for Kras-mutated lung and pancreatic cancer progression.

**Figure 4:**
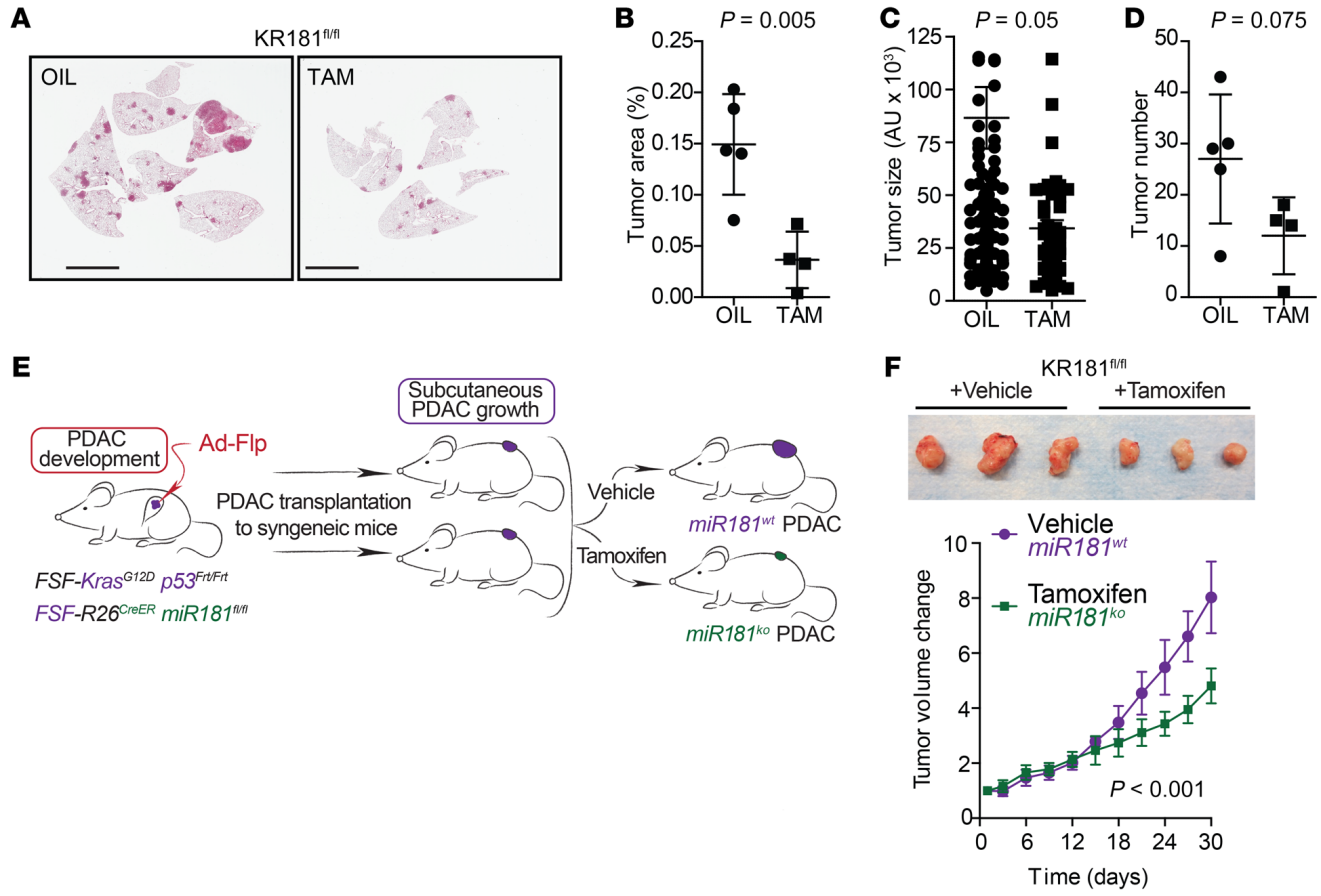
Mutant-*Kras* lung and pancreatic cancer progression is dependent on miR181ab1 expression. (**A**) Representative H&E-stained sections of vehicle- and tamoxifen-treated KR181^fl/fl^ lungs. Scale bars: 5 mm. (**B**) Tumor area in KR181^fl/fl^ mice treated with vehicle (oil, *n* = 5) or tamoxifen (*n* = 4) compared by *t* test. (**C**) Average tumor size in KR181^fl/fl^ vehicle- (*n* = 135) and tamoxifen-treated (*n* = 48) groups compared by median test. (**D**) Mean number of tumors per mouse in KR181^fl/fl^ vehicle- and tamoxifen-treated mice compared by *t* test. (**E**) Schematic representation of experiment. Ad-Flp, adenoviral FLP recombinase. (**F**) Representative images of xenografted *Kras*-mutated PanIN from KFR181^fl/fl^ mice treated with vehicle (*n* = 8) or tamoxifen (*n* = 8) (upper panel) and bar graph of the average of tumor size in each group (lower panel) compared by *t* test.

To assess the role of miR181ab1 in tumor maintenance, we turned to a model system that allows for the deletion of the *Mir181ab1* cluster in already established cancers. The *Kras*^LA2-G12D/+^ allele spontaneously recombines to initiate lung tumors (4). We crossed these mice to the *Rosa26*^CreERT2/+^ *Mir181ab1*^fl/fl^ mice to generate KR181^fl/fl^ mice in which whole-body deletion of floxed *Mir181ab1* alleles occurs upon tamoxifen administration (48). At 8 weeks of age, when adenomas are already spontaneously developing in the lungs (4), KR181^fl/fl^ mice were given tamoxifen or vehicle for 1 week. Lungs were harvested 8 weeks after the last dose of tamoxifen and histologically analyzed. A significant decrease in the average tumor burden of KR181^fl/fl^ mice treated with tamoxifen was observed (Figure 4, A and B), with a reduction in lung tumors size and number (Figure 4, C and D). Therefore, depletion of the *Mir181ab1* cluster not only interferes with tumor initiation but also affects the development of established tumors, nominating the transcriptional regulon of this cluster as a potential therapeutic target in this disease.

We also investigated whether miR181ab1 is required for pancreatic cancer progression and maintenance by generating *Kras*^FSF-G12D/+^ *Trp53^Frt/Frt^ Rosa26*^CreERT2/+^ *Mir181ab1*^fl/fl^ (KPR181^fl/fl^) mice. In these mice, activation of the *Kras* oncogene, deletion of *Trp53*, and expression of CreER is achieved by administration of an adenovirus expressing the FLP recombinase (adFlp) directly into the pancreatic parenchyma (49). KPR181^fl/fl^ mice were administered adFlp to initiate tumorigenesis. Upon PDAC development (~50 days after infection) tumors were harvested and allografted into immunodeficient mice. Ablation of *Mir181ab1* in this model is achieved by intraperitoneal injection of tamoxifen, which triggers CreER recombination of the *Mir181ab1*^fl/fl^ alleles (Figure 4E). *Mir181ab1* deletion resulted in decreased tumor volume (Figure 4F), indicating an important role for miR181ab1 in established-PDAC growth.

### Mir181ab1 loss in mutant-Kras cancer cells adversely impacts cell proliferation.

**Figure 5:**
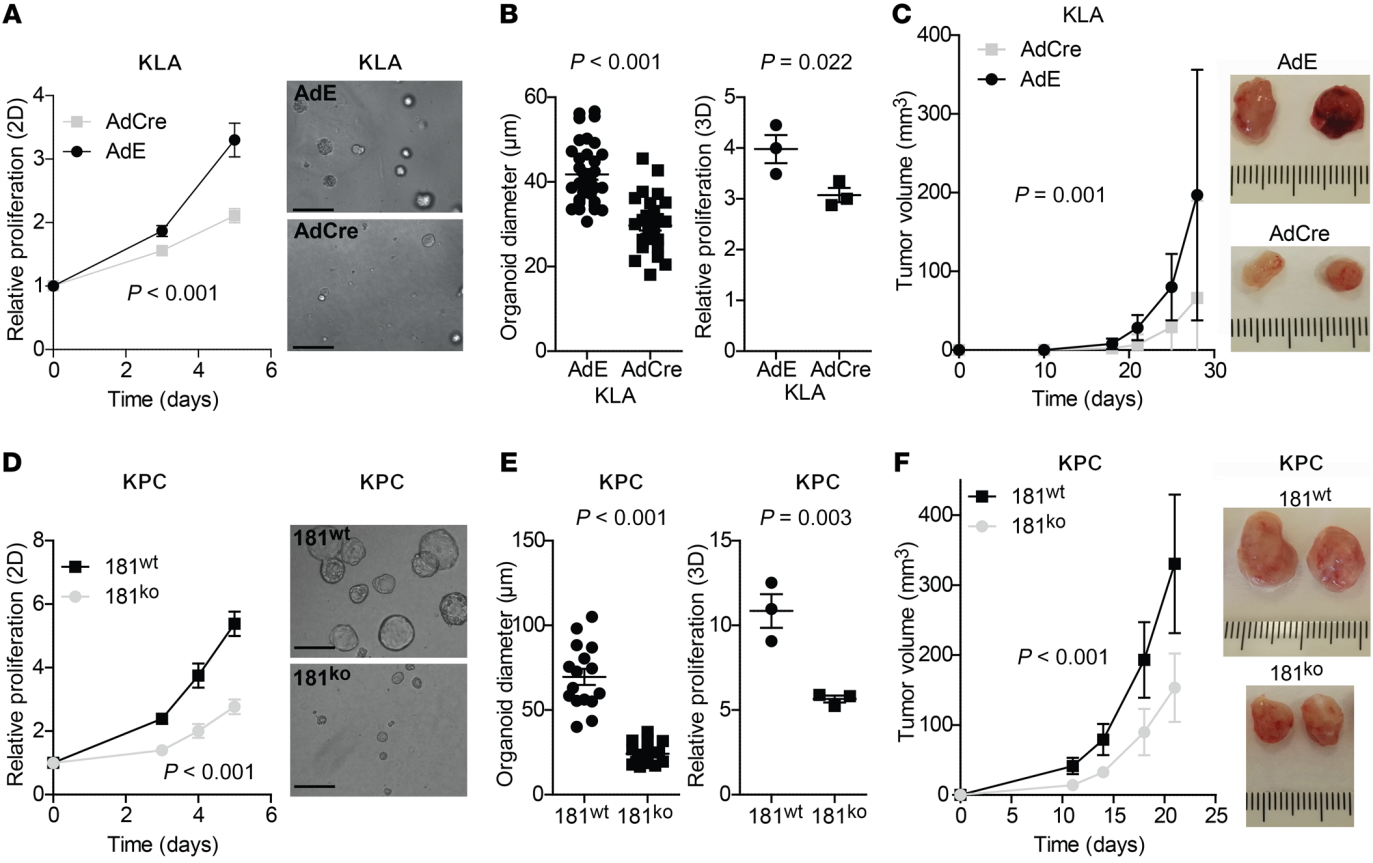
Effect of *Mir181ab1* loss in mutant *Kras*–driven cancer cells. (**A**) Cell proliferation of KLA cells, plated after 48 hours of adCre or adEmpty (adE) treatment, assessed by MTS (*n* = 5) and compared by *t* test. (**B**) 3D culture of KLA cells previously treated with adCre or adE for 48 hours. Left: Representative KLA organoid images on day 4 after seeding. Scale bars: 100 μm. Middle: KLA organoid size quantification on day 4 after seeding (*n* = 29–45) compared by *t* test. Right: Proliferation of KLA organoids measured by CellTiterGLO (*n* = 3) and compared by Mann-Whitney *U* test. (**C**) Left: Average tumor volume of allografts from mouse KLA cells previously treated with adE or adCre for 48 hours (*n* = 6 per group) and compared by *t* test. Right: Representative images of KLA tumors in the presence and absence of *Mir181ab1*. (**D**) Cell proliferation of KPC miR181^wt^ and miR181^ko^ cells assessed by MTS (*n* = 6) and compared by *t* test. (**E**) Left: Representative images of KPC miR181^wt^ and miR181^ko^ organoids on day 4. Scale bars: 100 μm. Middle: Organoid size quantification on day 4 after seeding (*n* = 16) and compared by *t* test. Right: Proliferation of KPC miR181^wt^ and miR181^ko^ organoids measured by CellTiterGLO (*n* = 3) and compared by Mann-Whitney *U* test. (**F**) Left: Average tumor volume of allografts from mouse KPC miR181^wt^ and miR181^ko^ cells (*n* = 8 per group) and compared by *t* test. Right: Representative images of KPC miR181^wt^ and miR181^ko^ tumors. Proliferation assays (**A**, **B**, **D**, and **E**) are representative of 3 independent experiments.

The above findings in multiple GEMMs of tumor initiation and progression indicate that the effect of miR181ab1 on mutant *Kras*–driven tumorigenesis is likely cell autonomous and not tissue specific. However, they do not rule out whether cancer cells can influence the surrounding microenvironment to foster tumor progression. To determine the role of miR181ab1 exclusively in cancer cells, cell lines were isolated from lung and pancreatic cancer mouse models. KLA cells derived from KR181^fl/fl^ mice carry the oncogenic-*KRAS* allele but are wild type for *Mir181ab1* until delivery of Cre using adenoviral infection (Supplemental Figure 2, A–C). Loss of *Mir181ab1* led to significant reduction in the number of cells (Figure 5A) and impaired cell growth in a 3D organoid assay (Figure 5B). Moreover, cells xenografted after *Mir181ab1* deletion generated smaller tumors (Figure 5C), paralleling the results obtained in the GEMM. Of note, the tumors that did develop retained expression of miR181a and miR181b due to incomplete recombination of the *Mir181ab1* allele (Supplemental Figure 2, D and E), suggesting selective pressure for the *Mir181ab1* cluster expression in oncogenic KRAS–driven tumors.

In mouse PDAC cell lines (KPC181^wt^ and KPC181^ko^) (Supplemental Figure 2F), analysis of the growth kinetics showed that KPC181^ko^ cells had a slower proliferation rate than KPC181^wt^ cells (Figure 5D). Moreover, growth of 3D organoids from KPC181^ko^ cells was much lower than the wild-type counterparts (Figure 5E). Lastly, KPC181^ko^ cells generated smaller tumors in immunodeficient mice than KPC181^wt^ ones (Figure 5F). Collectively, these data suggest that expression of both members of the *Mir181ab1* cluster favors a pro-oncogenic phenotype in epithelial lung and pancreatic cancer cells with *Kras* mutations.

### Dual miR181a and miR181b expression is necessary to rescue the Mir181ab1-loss phenotype.

**Figure 6:**
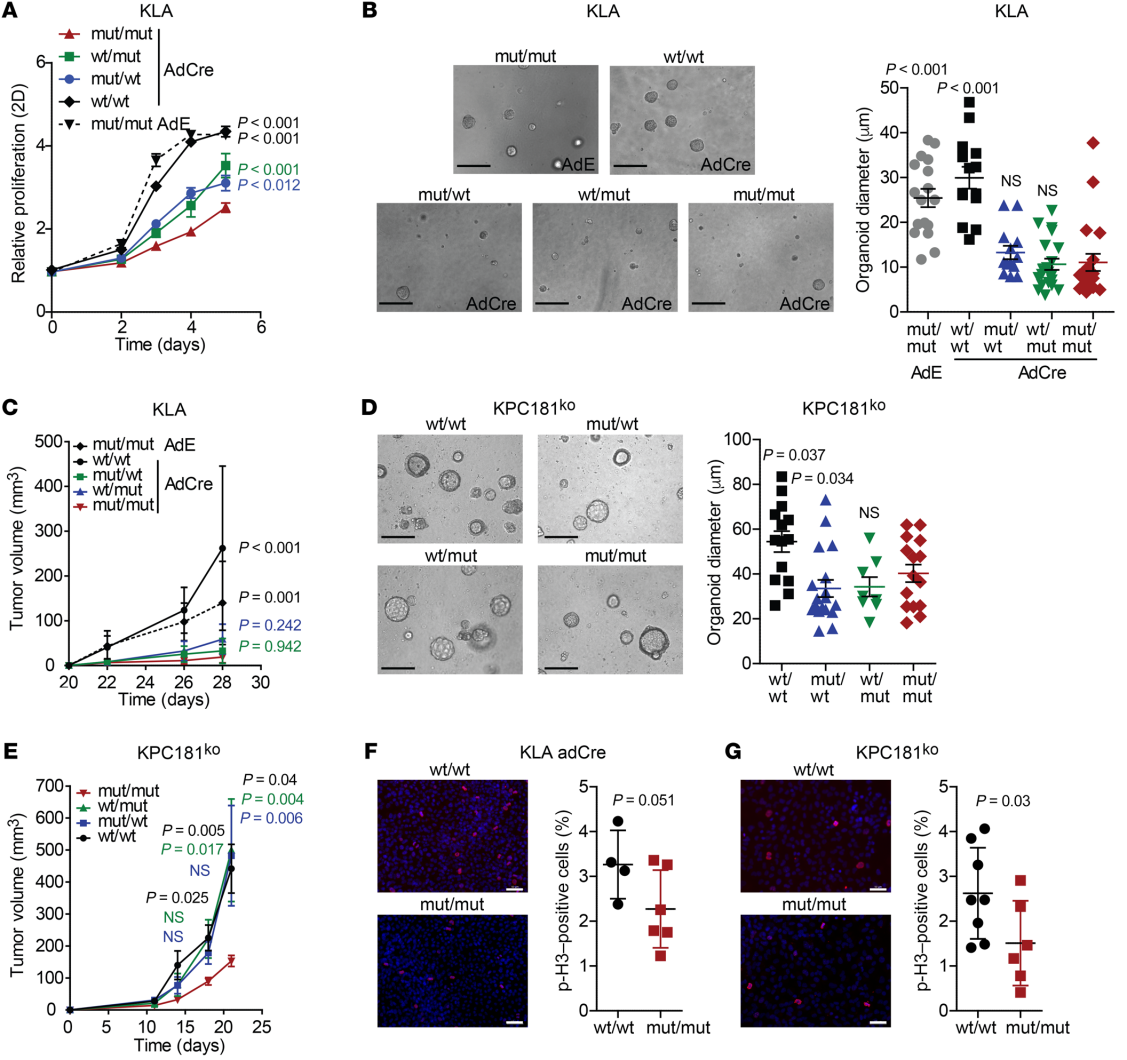
Simultaneous reconstitution of *Mir181a1* and *Mir181b1* rescues the *Mir181ab1-*knockout phenotype. (**A**) Cell proliferation of KLA cells transduced with retroviral vectors containing *Mir181a1* and *Mir181b1* genomic DNA and treated with adCre or adE for 48 hours assessed by MTS (*n* = 3). Analysis by ANOVA. wt/wt: wild-type seed sequence of *Mir181a1* and *Mir181b1*. mut/wt: mutated seed sequence of *Mir181a1* and wild-type sequence of *Mir181b1*. wt/mut: wild-type seed sequence of *Mir181a1* and mutated sequence of *Mir181b1*. mut/mut: mutated seed sequence of *Mir181a1* and *Mir181b1*. (**B**) 3D culture of KLA cells expressing the different *Mir181a1* and *Mir181b1* constructs. Left: Representative images of KLA miR181 organoids on day 4 after 48 hours of treatment with adE or adCre. Scale bars: 100 μm. Right: KLA miR181 organoid size quantification on day 3 after seeding (*n* = 14–20) and compared using ANOVA. (**C**) Average tumor volume of allografts from mouse KLA cells transduced with the different *Mir181a1* and *Mir181b1* constructs, previously treated with adE or adCre (*n* = 6 per group), assessed by ANOVA. (**D**) 3D culture of KPC miR181^ko^ cells transduced with the *Mir181a1* and *Mir181b1* constructs. Left: KPC miR181^ko^ organoids on day 4 after seeding. Scale bars: 100 μm. Right: Organoid size quantification on day 4 after seeding (*n* = 8–18) and compared by ANOVA. (**E**) Average tumor volume of allografts from mouse KPC miR181^wt^ and miR181^ko^ cells (*n* = 6 per group) and compared using ANOVA. (**F** and **G**) Phospho–histone H3 immunofluorescence images and analyses of wt/wt and mut/mut adCre–treated KLA cells (**F**), and in KPC181^ko^ wt/wt and mut/mut cells (**G**) 72 hours after seeding (*n* = 6–8). Results were compared by Kruskal-Wallis test.

The *Mir181ab1* cluster contains 2 miRNA genes, *Mir181a1* and *Mir181b1*. To dissect the contribution of each miRNA to the *Mir181ab1*-knockout phenotype, we took advantage of the cellular models to manipulate miR181a1 and miR181b1 expression. First, *Mir181a*, *Mir181b*, or both were transduced in the KLA cell line using retroviral vectors (38) (Supplemental Figure 3A). These vectors included the genomic region spanning *Mir181ab1* on chromosome 1 yet differed in that the seed sequence binding the mRNA’s 3′UTR is wild type in *Mir181a1* and *Mir181b1* (wt/wt), mutated in *Mir181a1* (mut/wt), mutated in *Mir181b1* (wt/mut), or mutated in both (mut/mut), abrogating single- or dual-miRNA function. Reconstitution of both miR181a1 and miR181b1 rescued the cell proliferation rate impaired by the cluster deletion, whereas individual expression of each miRNA was unable to fully recover the normal phenotype (Figure 6A). Likewise, only simultaneous expression of miR181a1 and miR181b1 successfully recovered cell growth in 3D (Figure 6B and Supplemental Figure 3B) and in a xenograft model (Figure 6C and Supplemental Figure 3C).

The miR181 constructs were overexpressed in pancreatic cancer cells (Supplemental Figure 3D). Dual expression of miR181a1 and miR181b1 enhanced organoid growth in 3D assays compared with miR181ab1-deficient cells (Figure 6D and Supplemental Figure 3E). Additionally, combined miR181a1 and miR181b1 expression yielded significantly larger tumors in a xenograft model at the earlier time points (15 and 18 days), although this growth advantage was lost to miR181a1- or miR181b1-overexpressing cells at the end of the experiment (day 22) (Figure 6E and Supplemental Figure 3F). Mouse lung and pancreatic cancer cells expressing miR181ab1 underwent mitosis more efficiently than miR181ab1-deficient cells (Figure 6, F and G). Consistent with these results, combined miR181a1 and miR181b1 overexpression shortened progression time through cell cycle of both mouse lung and pancreatic cancer cells, indicative of an increased proliferation, as shown by a significant percentage of cells reaching G2/M phase with regard to *Mir181ab1*-knockout cells (Supplemental Figure 3, G and H). Taken together, these observations support the idea that both miR181a1 and miR181b1 are necessary for proficient induction of tumorigenesis by mutant *Kras*, in part by regulating cell cycle progression.

### Mir181ab1 expression enhances proliferation of lung and pancreas epithelial cells.

To investigate the potential role of miR181ab1 in human cancer, we queried its association with oncogenic *KRAS* expression in vitro. For these experiments, we used immortalized bronchial epithelial cells (3KT), wild-type (H2126), and mutant-*KRAS* (H1792) lung cancer cells (Supplemental Figure 4A). Upregulation of both miR181a and miR181b in wild-type *KRAS* lung cancer cells was observed upon expression of oncogenic *KRAS* (Supplemental Figure 4, B and C). 3KT cells transduced with mutant *KRAS* proliferated faster than control cells in 2D and 3D (Supplemental Figure 4, D and E).

**Figure 7:**
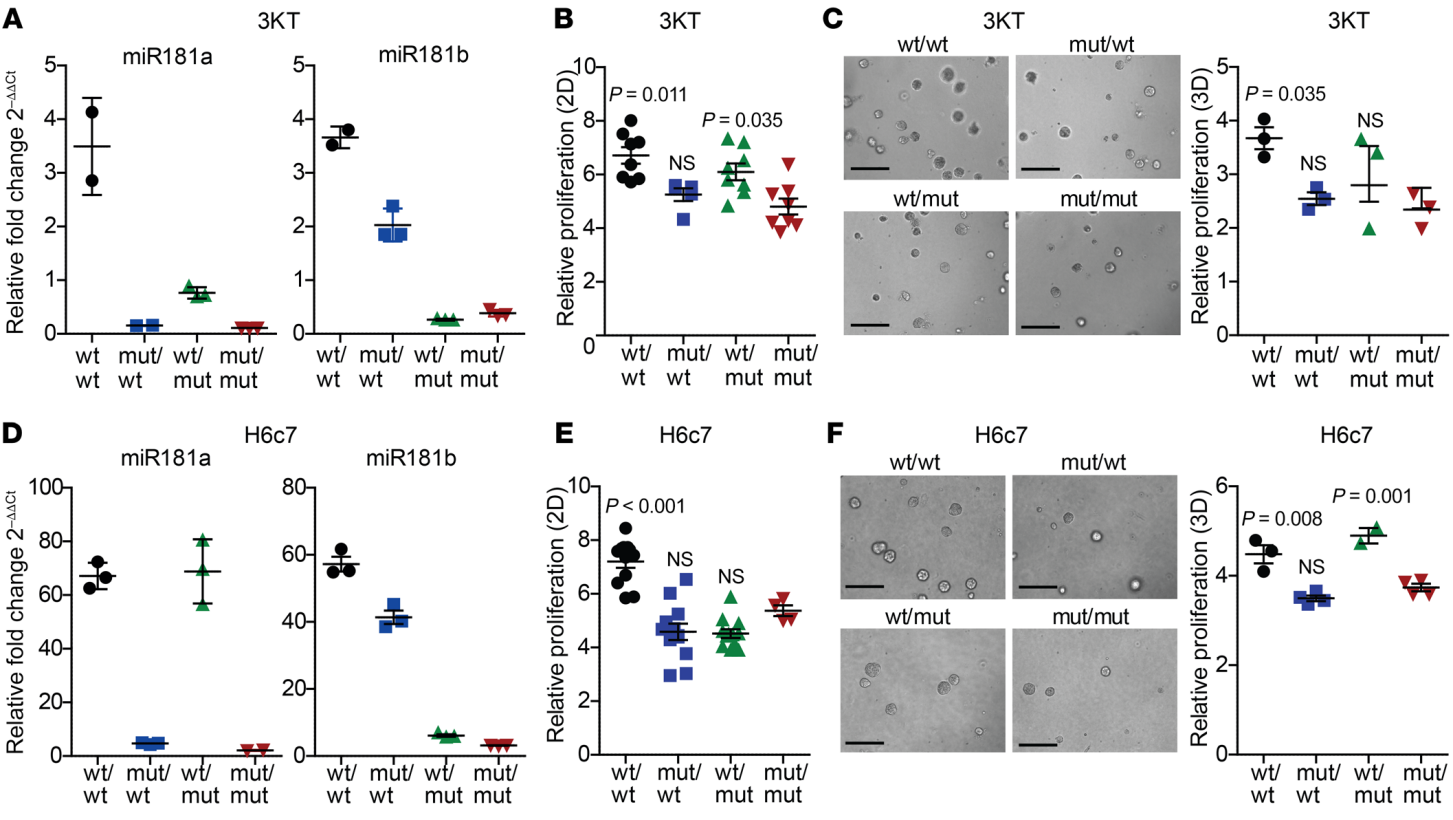
miR181ab1 promotes 2D and 3D proliferation in lung and pancreas epithelial cells. (**A**) miR181a and miR181b expression by quantitative PCR in 3KT cells transduced with the different *Mir181a1* and *Mir181b1* constructs (*n* = 3). (**B**) Cell proliferation of the same cells as in **A** assessed by MTS (*n* = 5–8) and compared using ANOVA. (**C**) 3D culture of the same cells as in **A**. Left: Representative images of organoids on day 4. Scale bars: 100 μm. Right: Proliferation of organoids measured by CellTiterGLO on day 4 relative to day 1 after seeding (*n* = 3) and compared by ANOVA. (**D**) miR181a and miR181b expression in human pancreatic ductal cells (H6c7) transduced with the different *Mir181a1* and *Mir181b1* constructs (*n* = 3). (**E**) Cell proliferation of the same H6c7 cells as in **D** assessed by MTS (*n* = 4–12) and compared by ANOVA. (**F**) 3D culture of the same H6c7 cells as in **D**. Left: Representative images of organoids on day 3. Scale bars: 100 μm. Right: Proliferation of organoids measured by CellTiterGLO on day 4 relative to day 1 after seeding (*n* = 3) and compared by ANOVA and Dunnett’s multiple-comparisons test.

Next, we investigated if miR181ab1 plays a role in malignant transformation. First, we constructed immortalized lung 3KT cells expressing *Mir181a1*, *Mir181b1*, or both (Figure 7A). Simultaneous overexpression of both miRNAs increased proliferation of 3KT cells, while each individual miRNA had little or no effect (Figure 7B). Likewise, only combined overexpression of miR181a1 and miR181b1 enhanced growth of 3KT cells in 3D cultures compared with single miRNA overexpression (Figure 7C and Supplemental Figure 4F). These results were partially recapitulated in immortalized human pancreatic ductal epithelial cells (H6c7) transduced with the miR181 constructs (Figure 7D). In these cells, dual miR181a1 and miR181b1 overexpression induced the highest proliferation rate among the different constructs in 2D cultures (Figure 7E). Interestingly, in 3D cultures the effect of the combined miRNA overexpression was similar to that of miR181a expression alone (Figure 7F and Supplemental Figure 4G), suggesting distinct functional relevance of these 2 miRNAs depending on growth conditions.

### MiR181ab1 plays a functionally relevant role in human oncogenesis.

**Figure 8:**
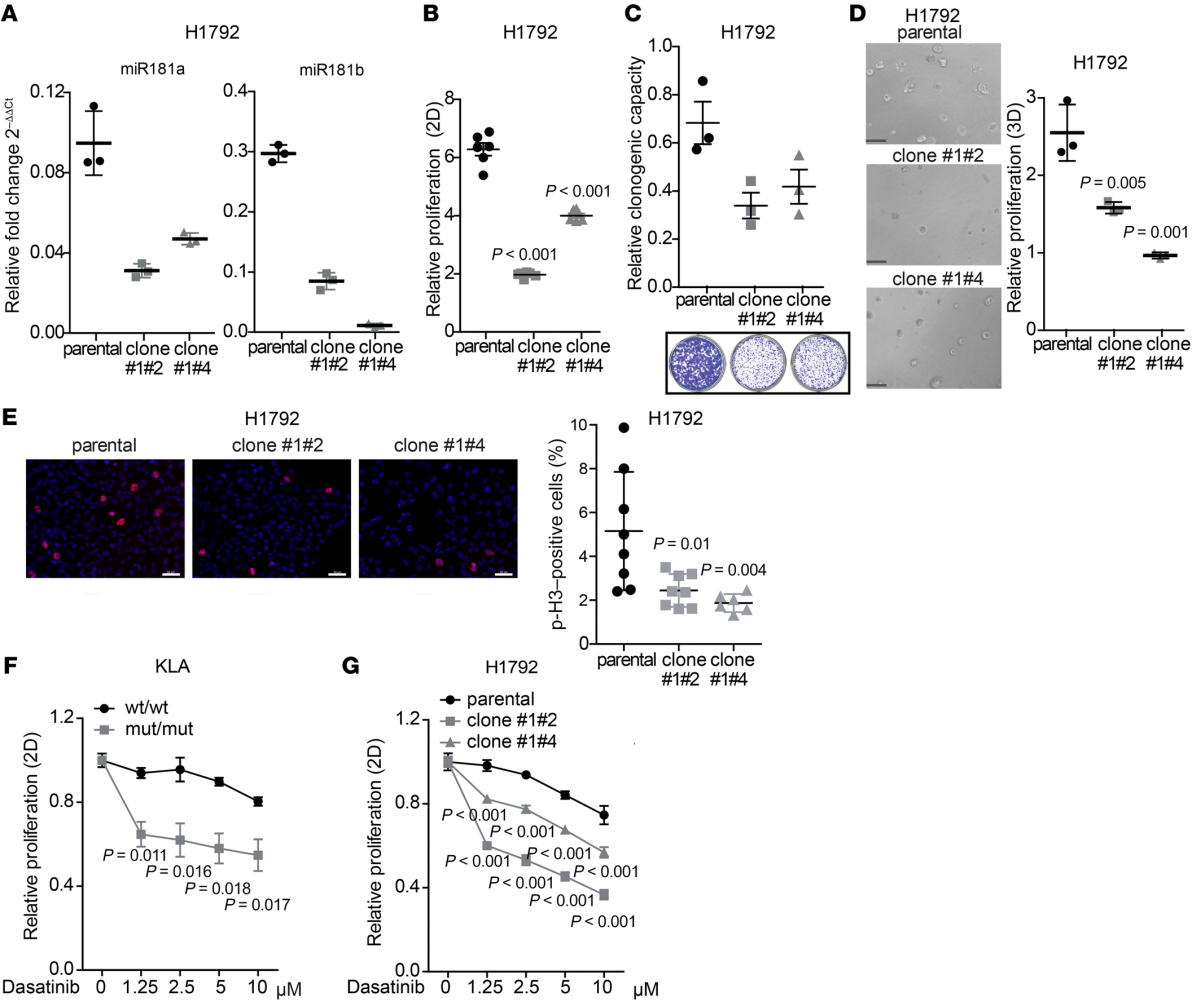
A functional role for miR181ab1 in human cancer. (**A**) miR181a and miR181b expression assessed by quantitative PCR of control and partially CRISPRed knockout clones for mir181ab1 (clones #1#2 and #1#4) of human lung cancer cells (H1792) (*n* = 3). (**B**) Cell proliferation of H1792 control and mir181ab1-CRISPRed clones assessed by MTS 3 days after seeding (*n* = 6) and compared by ANOVA. (**C**) Clonogenic ability of H1792 control and mir181ab1-CRISPRed clones. Top: Relative absorbance of dissolved crystal violet on day 10 (*n* = 3) was compared by ANOVA. Bottom: Representative images of H1792 parental and mir181ab1-CRISPRed clones on day 10. (**D**) 3D culture of parental and mir181ab1-CRISPRed H1792 clones. Left: Representative images of organoids on day 4. Scale bars: 100 μm. Right: Proliferation of organoids measured by CellTiterGLO on day 4 relative to day 1 after seeding (*n* = 3) was compared by ANOVA. (**E**) Phospho–histone H3 immunofluorescence images and analyses of H1792 control and mir181ab1-CRISPRed clones (*n* = 7–8). Results were compared by Brown-Forsythe test. (**F**) wt/wt and mut/mut adCre–administered KLA cells treated with dasatinib for 72 hours at indicated doses (*n* = 4). Results are relative to nontreated control cells and were compared by *t* test. (**G**) H1792 control and mir181ab1-CRISPRed clones (clones #1#2 and #1#4) treated with dasatinib for 72 hours at indicated doses (*n* = 4) and compared by ANOVA.

To determine if miR181a1 and miR181b1 influence homeostasis of mutant-*KRAS* cancer cells, we used a CRISPR/Cas9-based knockout strategy using sgRNAs flanking the genomic region spanning the *MiR181ab1* cluster (Supplemental Figure 5A). Clonal expansion of CRISPR-engineered lung cancer cells (H1792) was used to isolate 2 clones with partial knockout of the cluster (clones #1#2 and #1#4) (Supplemental Figure 5B) that led to greater than 50% decreased expression of both miR181a and miR181b (Figure 8A). Of note, proliferation of parental cells and single cell–derived wild-type clones was very similar (Supplemental Figure 5C). Partial deletion of the *MiR181ab1* cluster decreased proliferation, colony formation ability, and 3D growth (Figure 8, B–D, and Supplemental Figure 5D). These findings were associated with a decreased percentage of mitotic cells in partially *MiR181ab1* cluster–knocked out cells compared with wild-type ones (Figure 8E) as well as a delayed cell cycle progression, evidenced by a larger percentage of control cells reaching G1 phase (Supplemental Figure 5E). Consistent with a critical role for miR181ab1, we were unable to obtain clones with full knockout (data not shown). Indeed, subsequent reintroduction of sgRNAs in the 2 partial knockout clones yielded no clones with full abrogation of miR181ab1, although analysis of these pools of cells did reveal greater and more efficient knockout of the cluster (Supplemental Figure 5F). Overall, these results suggest that the *MiR181ab1* cluster is required for maintenance of the oncogenic phenotype in *KRAS*-driven human non–small cell lung cancer.

Effective targeting of mutant-KRAS tumors likely requires concomitant inhibition of different effector pathways. To ascertain whether miR181ab1 inhibition would enhance the effect of targeted therapies, we screened a series of inhibitors available in the clinic or in late clinical phases. Murine lung cancer cells lacking the *Mir181ab1* cluster were more sensitive to the multiple–tyrosine kinase (BCR-ABL, SRC, c-KIT) inhibitor dasatinib than those cells in which *Mir181ab1* was reconstituted (Figure 8F). These results were recapitulated in a human lung cancer cell line expressing oncogenic KRAS where *MiR181ab1* was partially knocked out (Figure 8G). Taken together, these observations suggest that miR181ab1 plays an important role in human *KRAS*-mutated oncogenesis and that its ablation could cooperate with targeted agents to improve therapeutic efficacy in *KRAS*-mutated cancers.

### MiR181ab1 expression is regulated by TGF-β in mutant-KRAS lung and pancreatic cancer cells.

To determine the molecular mechanisms of *Mir181ab1* regulation, we first evaluated the role of specific effector pathways using pharmacological inhibition in mouse lung and pancreatic cancer cells. miR181a and miR181b expression levels were assessed 3 and 12 hours after inhibition. Effector inactivation did not decrease either miR181a or miR181b levels, suggesting that *Mir181ab1* is not directly regulated through these effectors by KRAS (Supplemental Figure 6, A and B).

**Figure 9:**
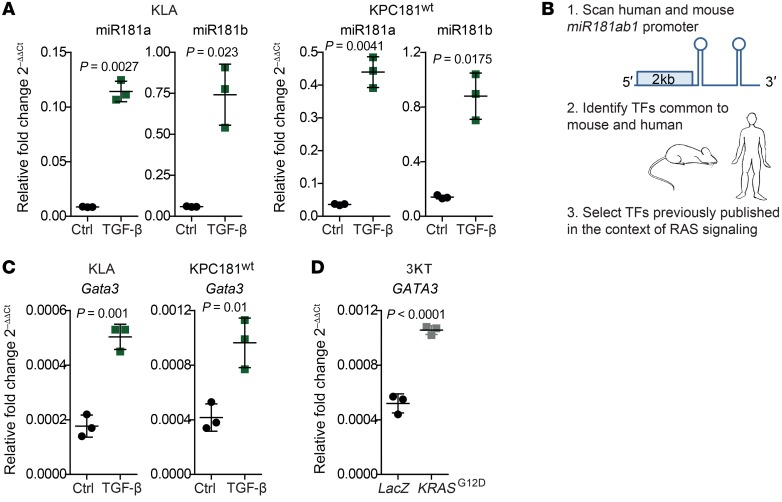
Regulation of the *Mir181ab1* cluster. (**A**) miR181a and miR181b expression assessed by quantitative PCR in mouse lung cancer (KLA) and pancreatic cancer (KPC) cells treated with 10 ng/mL TGF-β for 3 hours (*n* = 3) and compared by *t* test. (**B**) Schematic representation of the strategy to unveil transcription factors potentially regulating miR181ab1 expression. (**C**) *Gata3* expression assessed by quantitative PCR in the same cell lines as in **A** (*n* = 3) compared by *t* test. Error bars correspond to SD. (**D**) *GATA3* expression assessed by quantitative PCR in 3KT cells expressing a control gene (*LacZ*) or a mutated version of *KRAS* (G12D) (*n* = 3) compared by *t* test.

As an additional means of *Mir181ab1* regulation, we explored the potential involvement of TGF-β, a growth factor previously described to increase miR181a and miR181b expression in hepatocellular carcinoma (50). Of note, miR181a and miR181b expression was upregulated 3 hours after exogenous addition of TGF-β in both mouse lung and pancreatic cancer cells (Figure 9A). These results suggest that the TGF-β signaling cascade could be involved in *Mir181ab1* regulation in both tumor types.

To ascertain potential transcriptional regulators of the *Mir181ab1* cluster, we carried out a 3-step analysis (Figure 9B). First, we scanned a 2-kb region of the promoter of the mouse and human *MiR181ab1* gene to uncover transcription factors (TFs) binding to specific motifs in this genomic region. Next, we identified those TFs that are conserved across species. Lastly, we focused on those TFs that had been previously linked to RAS signaling (*Cebpα*, *Cebpβ*, *Cmyb*, *Evi1*, *Meis1*, *Gata2*, *Gata3*, and *Foxa2*) (51–58). Quantitative PCR (qPCR) analysis of the TFs revealed upregulation of *Gata3* in both mouse lung and pancreatic cancer cell lines after TGF-β treatment for 3 hours (Figure 9C), while no consistent upregulation in the 2 cell lines was found for the remaining TFs (Supplemental Figure 6, C and D).

The similar expression pattern of *Gata3* and miR181a/miR181b, and the presence of regulatory elements in the *Mir181ab1* promoter suggested that miR181a1 and miR181b1 could be regulated by *Gata3*. To substantiate this potential association, further analysis of *GATA3* expression was done in immortalized lung epithelial cells expressing mutant *KRAS* in which miR181a and miR181b levels increased upon oncogene expression (Supplemental Figure 4C). The results showed that *GATA3* is also overexpressed upon oncogenic KRAS expression (Figure 9D). These findings suggest that GATA3 upregulation by the *KRAS* oncogene may mediate miR181a and miR181b expression.

### A miR181ab1 signature predicting poor prognosis in KRAS-driven cancers includes genes with a tumor-suppressive role.

**Figure 10:**
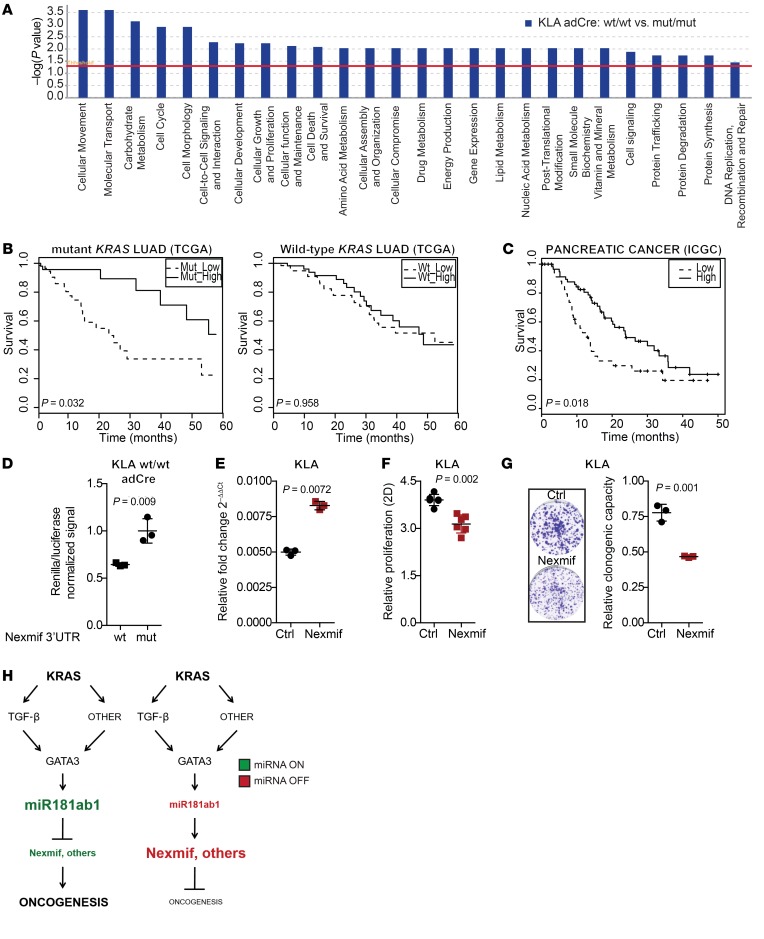
miR181ab1 targets involved in human KRAS-driven cancer. (**A**) Graph representing biological processes enriched in KLA wt/wt with regard to KLA mut/mut adCre–treated cells by Ingenuity Pathways Analysis (IPA). (**B**) Kaplan-Meier plots of lung adenocarcinoma (LUAD) patients from TCGA stratified based on median expression of the 10-gene miR181ab1 signature (log-rank test). Left: Mutant-*KRAS* LUAD patients. Right: Wild-type KRAS *LUAD* patients. Putative miR181ab1 targets in human cancer were selected if a seed sequence was predicted to exist in the 3′UTR by at least 3 prediction algorithms. (**C**) Kaplan-Meier plot of pancreatic ductal adenocarcinoma (PDAC) patients from ICGC based on the 10-gene miR181ab1-target signature (log-rank test). (**D**) Luciferase assay of KLA wt/wt adCre–treated cells that were transfected with a psiCheck vector encoding the wild-type functional 3′UTR of *Nexmif* or a seed-mutated (mut) version that impedes miR181ab1 binding (*n* = 3). Renilla results are normalized to firefly luciferase signal and compared by *t* test. (**E**) *Nexmif* expression assessed by quantitative PCR in control- (GFP) and *Nexmif*-overexpressing KLA cells (*n* = 3) compared by *t* test. (**F**) Cell proliferation analysis by MTS of control- (GFP) and *Nexmif*-overexpressing KLA cells (*n* = 6) compared by *t* test. (**G**) Clonogenic analysis of the same cells as in **F** (*n* = 3) compared by *t* test. (**H**) Proposed model of miR181ab1 regulation and function in the context of mutant-*KRAS* oncogenesis.

Next, to define key miR181ab1 targets we first evaluated protein expression levels of KRAS and RASSF1A, previously reported as miR181 targets (59, 60). No differential expression of either KRAS or RASSF1A was observed upon genetic *MiR181ab1* manipulation in our mouse and human cellular systems (Supplemental Figure 7, A and B), suggesting no direct involvement in the *MiR181ab1* loss-of-function phenotype. We then undertook an unbiased approach to identify potential miR181ab1 targets. RNA sequencing was performed on mouse lung cancer cells (KLA) expressing wild-type (wt/wt) or seed-mutated (mut/mut) versions of *Mir181ab1*. Both cell lines were treated with adCre to deplete endogenous miR181a1 and miR181b1 in order to obtain homogeneous cell pools for comparison because single-cell qPCR revealed that expression of miR181a and miR181b is highly heterogeneous in the parental cell pool (Supplemental Figure 8, A and B). The heterogeneous expression observed is consistent with previous studies reporting heterogeneous expression of this miRNA in cancer cell populations of distinct tissue types (50, 61, 62). A list of 111 differentially expressed genes (54 downregulated and 57 upregulated) was obtained (B > 0 and logFC > 0.5 or < 0.5) (Supplemental Figure 8C and Supplemental Table 1) and queried for molecular functions using Ingenuity Pathway Analysis (IPA). The top 10 processes associated with this gene list are cellular movement, molecular transport, carbohydrate metabolism, cell cycle, cell morphology, cell-to-cell signaling and interaction, cellular development, cellular growth and proliferation, cellular function and maintenance, and cell death and survival (Figure 10A). These findings are consistent with data above indicating that miR181ab1 regulates cell proliferation and cell cycle progression.

Next, we focused on those genes whose expression decreased upon exogenous reconstitution of miR181ab1, as they could include putative direct targets of the miRNA cluster. First, the list of downregulated genes was queried against the Molecular Signature Database (MSigDB; https://www.gsea-msigdb.org/gsea/msigdb/index.jsp) to search for miRNAs involved in the regulation of this gene set. The top miRNA predicted to regulate genes in the downregulated list was the miR181 family (Supplemental Table 2), suggesting that our reconstitution approach provides an optimal model to unveil *MiR181ab1* direct targets. The expression decrease of these *MiR181ab1* putative targets (*C77370/Kiaa2022/Nexmif*, *Fbxo33*, *Meaf6*, *Med8*, *Mfsd6*, *Plekhj1*, *Rbbp7*, and *Scoc*) was validated by qPCR in independent samples (Supplemental Figure 8D). Review of the known activity of the proteins encoded by these genes provides a potential mechanism for the effect of *MiR181ab1* in *KRAS*-driven oncogenesis. For example, *Fbx033* is known to promote degradation of the oncoprotein YB-1 (63), and *Rbbp7* has been reported to function similarly to the Ras negative regulator MSI1 in yeast (64), suggesting an overall tumor-suppressive function.

Next, to test the clinical relevance of miR181ab1 targets in mutant-*KRAS* patients, we unveiled an accurate list of putative targets in human cancer. To do this, we used a conservative approach by identifying genes for which a seed sequence in the 3′UTR was predicted to exist by at least 3 independent prediction algorithms (65). This analysis yielded a 10-gene set of downregulated genes with a seed sequence predicted to be bound by miR181a1 or miR181b1 and consisted of *NEXMIF*, *DEK*, *DTX4*, *FBXO33*, *MEAF6*, *MED8*, *MFSD6*, *PLEKHJ1*, *RBBP7*, and *SCOC* (Supplemental Table 3). The 10-gene set was interrogated against the human lung cancer data set (The Cancer Genome Atlas [TCGA]; https://www.cancer.gov/about-nci/organization/ccg/research/structural-genomics/tcga) and pancreatic cancer data set (International Cancer Genome Consortium [ICGC]; https://icgc.org/). Low expression levels of the 10-gene set were associated with poor survival in lung cancer (lung adenocarcinoma [LUAD]) patients harboring *KRAS* mutations (*P* = 0.035), whereas no association was found in wild-type *KRAS* LUAD patients (*P* = 0.958) (Figure 10B). A similar trend was obtained in the analysis of pancreatic cancer (pancreatic ductal adenocarcinoma [PDAC]) patients, where low expression of the putative miR181ab1 targets was a marker of poor prognosis (*P* = 0.018) (Figure 10C). Collectively, these data suggest that miR181ab1 regulates a series of genes involved in the induction of the tumor phenotype whose expression associates with lung and pancreatic cancer patients’ survival, in line with its strong functional role in both types of mutant *KRAS*-driven cancers.

To ascertain the role of the identified genes as direct miR181ab1 targets, we focused on *Nexmif*, whose expression was largely decreased upon miR181ab1 overexpression. First, luciferase assays in miR181ab1-proficient mouse lung cancer cells were performed. Mutation of the miR181ab1 seed sequence in the 3′UTR of *Nexmif* led to an enhanced signal due to impaired miRNA binding (Figure 10D). Next, the functional implication of *Nexmif* was queried through ectopic expression in mouse lung cancer cells (Figure 10E). Overexpression of *Nexmif* significantly reduced cell proliferation and clone-forming capacity (Figure 10, F and G), consistent with a predicted tumor-suppressive role of miR181ab1 targets. Lastly, the clinical value of NEXMIF was investigated in human LUAD and PDAC data sets. Low levels of *NEXMIF* expression associated with a worse survival outcome in LUAD patients with *KRAS* mutations (Supplemental Figure 8E), with a similar trend observed for PDAC patients (Supplemental Figure 8F). Furthermore, NEXMIF expression was lower in LUAD patients harboring *KRAS* mutations (Supplemental Figure 8G).

Collectively, our data indicate that miR181ab1 is a KRAS effector with functional and clinical implications in *KRAS*-mutated lung and pancreatic tumorigenesis, whose expression regulation may rely on noncanonical KRAS downstream pathways. A proposed model for miR181ab1 regulation and function in the context of *KRAS* mutations is illustrated in Figure 10H.

## Discussion

*KRAS* is a key oncogene in the development of lung and pancreatic cancer. Understanding how KRAS signaling leads to gene expression changes that ultimately result in tumorigenesis is of paramount importance for the development of effective therapies. Given the complexity of the effector output downstream of KRAS, it is likely that small changes in the expression of multiple proteins can impact the relative strength of this output and thus strongly regulate oncogenesis. Here we demonstrate that miR181ab1 is a critical mediator of KRAS oncogenic effects in mouse and human. Although alterations such as gene amplification, deletion, and translocation are common events in genomic regions hosting miRNAs and can influence their expression in cancer (66), direct regulation by oncogenes and tumor suppressors can also significantly influence miRNA expression levels (27–31). Previous studies have suggested upregulation and functional roles for miR21, miR450b-5p, and miR30c in response to oncogenic KRAS in cancer (14–17). Here we used primary MEFs with conditional expression of oncogenic KRAS (36) to identify key dysregulated miRNAs. This approach likely identifies different miRNAs compared with overexpression of KRAS, which is known to lead to a distinct outcome in primary cells. Having first identified miR181 RNAs in MEFs, we then used several genetically engineered mouse models to determine the phenotype of loss of function of the *Mir181ab1* cluster in epithelial cells of the lung or pancreas. These studies convincingly demonstrate a key role for mir181ab1 in regulating the pro-oncogenic transcriptional output of oncogenic KRAS, a finding with potentially profound implications for the search for novel approaches to target KRAS-mutant cancers.

miR181 was first described as an miRNA preferentially expressed in B lymphoid cells of mouse bone marrow (67), and subsequent studies underscored a role for the *Mir181ab1* cluster in natural killer T cell development and T cell homeostasis (38, 40, 41). In cancer, the miR181 family is upregulated in several cancer types including T cell acute lymphoblastic leukemia (T-ALL) (68), pancreatic cancer (43, 44), and high-risk neuroblastoma (69). Moreover, early studies in T-ALL, hepatocellular carcinoma, and breast cancer demonstrated a potential functional oncogenic role for the miR181 family in cancer (38, 50, 61, 62). However, members of the miR181 family have been reported as tumor suppressors in other cancers such as AML (70), colorectal cancer (71), and lung cancer (72), and *KRAS* was recently proposed as a direct target of miR181a in AML (73). Although tissue-specific differences may account for some of these discrepancies in miR181ab1 expression and function, the data described here unequivocally demonstrate that miR181ab1 functions as a pro-oncogenic miRNA in lung and pancreatic tumors in which the *KRAS* oncogene is expressed. The *MiR181ab1* cluster is highly conserved across human and mouse species. Genetic ablation of this cluster in mouse and human models showed a consistent deleterious phenotype via a similar cellular mechanism, involving regulation of cell cycle, strongly favoring a conserved mechanism of action across species. Moreover, our results provide evidence suggesting that the main contributor of the miR181 family to the tumor phenotype induced by mutant KRAS is miR181ab1, similarly to what have been reported previously in a GEMM of Notch-induced T-ALL (38). The differential expression of the different miR181 clusters may be explained by the presence of distinct transcriptional regulatory elements in the promoter region of each cluster. Moreover, regulation of *Mir181ab1* by specific transcriptional regulators, such as GATA3, may require the action of noncanonical KRAS downstream pathways involving TGF-β activity. Nonetheless, genetic inhibition of the remaining 2 miR181 clusters, *Mir181ab2* and *Mir181cd*, would be required to definitively resolve their functional implication in mutant *KRAS*–driven oncogenesis. More importantly, despite the fact that the cellular systems deployed in this study suggest a cell-intrinsic effect of miR181ab1, further analyses to investigate how miR181ab1-depleted cells may influence other cell types to foster tumor formation and progression may shed more light on the mechanism of action of this miRNA cluster in KRAS-driven oncogenesis.

Mechanistically, we provide data indicating that expression of both members of the *Mir181ab1* cluster, *Mir181a1* and *Mir181b1*, is necessary to induce a complete oncogenic phenotype in *KRAS*-mutated tumors. Our current findings suggest a nonredundant function of miR181a1 and miR181b1; however, they do not completely rule out the possibility that both resident miRNAs may be needed to generate a threshold level of miR181 RNA required for the full oncogenic phenotype, especially given that they target the same 3′UTR sequence.

We also provide functional evidence that the miR181ab1 target *Nexmif* (*KIAA2022/KIDLIA*) has a functional role in *KRAS*-mutated tumors. NEXMIF is a nuclear protein that has been implicated in N-cadherin and δ-catenin signaling (74). To our knowledge, this gene has not previously been implicated in KRAS signaling or in oncogenesis. It is likely that miR181ab1 functions to dysregulate a large number of genes and that together these affect KRAS oncogenesis. Among other miR181ab1 targets, *FBXO33* mediates degradation of the oncoprotein YB-1 upon apoptosis (63). Additionally, *RBBP7* polyubiquitinates HUWE1 (75), an E3 ubiquitin ligase that interacts with PCNA to alleviate replication stress (76), a type of stress that characterizes mutant-*KRAS* tumors (77). These findings suggest that miR181ab1 may sustain *KRAS* oncogene action in part by stabilizing the expression of cancer-promoting genes. Further efforts beyond this study will be required to address these molecular relationships.

Our studies identify miR181ab1 as a potentially novel molecular target whose inhibition could be exploited to treat lung and pancreatic cancer patients. This is particularly significant, as our data suggest that inhibition of this cluster is relevant not only to tumor initiation but also for tumor progression and maintenance. Furthermore, functional testing of miR181ab1’s function in other mutant-*KRAS* tumors beyond lung and pancreatic cancer would be highly interesting, as those could also benefit from direct inhibition of this miRNA cluster. Given the limited number of effective treatments for patients harboring *KRAS* mutations, strategies based on miR181ab1 inhibition could represent a valuable therapeutic approach. Indeed, we showed that combinatorial approaches involving concomitant inhibition of miR181ab1 and dasatinib administration yielded a larger antitumor response in mutant-*KRAS* tumors. In this regard, *Mir181ab1*-knockout mice as well as triple-knockout mice without *Mir181ab1*, *Mir181ab2*, and *Mir181cd8* mice are normal and viable (38), which suggests that at least inhibition of KRAS-driven tumors by targeting miR181ab1 could be achieved without significant toxicity. Efforts to develop miRNA inhibitors are currently underway and various miRNA therapeutics, including anti-oncomiRs, have reached phase I and II clinical trials (78). Although challenges related to effective route of administration, long-lasting action, and potential adverse reactions are yet to be addressed, miR181ab1 inhibition could represent a new paradigm for the treatment of *KRAS*-mutated tumors.

## Methods

Additional information can be found in the Supplemental Methods.

### Cell lines.

Early passage MEFs (P3–P4) from E13.5 embryos were used for experiments. Mouse lung cancer (KLA-KR181^fl/fl^) and pancreatic cancer (KPC181^wt^ and KPC181^ko^) cell lines were isolated from corresponding GEMMs. MEFs and mouse cancer cell lines were grown in DMEM supplemented with 10% FBS and 1% penicillin-streptomycin. Human non–small cell lung cancer cells used were either wild-type *KRAS* (NCI-H2126) or mutant *KRAS* (NCI-H1792), were grown in RPMI1640 supplemented with 10% FBS and 1% penicillin-streptomycin, and were acquired from ATCC. 3KT cells were a gift from John Minna (UTSouthwestern, Dallas, TX, USA) (79). 3KT and H6c7 cells (Kerafast Inc.) were grown in keratinocyte medium (GIBCO). Human cancer cell lines were authenticated by the Genomics Unit at Center for Applied Medical Research (CIMA) using Short Tandem Repeat profiling (AmpFLSTR Identifiler Plus PCR Amplification Kit, Thermo Fisher Scientific). Cell lines were tested with the MycoAlert Mycoplasma Detection Kit (LONZA). Only mycoplasma-negative cells were used.

### 3D cultures.

Previously published protocols for the generation of pancreatic cancer organoids were followed (80). Organoid images were obtained using a DMI3000 inverted microscope from Leica at ×10 magnification. Diameter quantification was done with ImageJ (NIH).

### Luminex-based miRNA profiling.

Characterization of miRNA profiles in wild-type and mutant-*Kras* MEFs was done following previously described protocols (37).

### Mouse work.

For lung cancer experiments, *Kras*^LSLG12D/+^ *Mir181ab1*^–/–^ mice were on a mixed 129/Sv and C57BL/6 background, whereas *Kras*^LSLG12D/+^ *Mir181ab1*^fl/fl^ mice were on a C57BL/6 background. *Kras*^LA2/+^ *Mir181ab1*^fl/fl^ *Rosa26Cre*^ERT2^ compound mice were on a mixed 129/Sv and C57BL/6 background. For pancreatic cancer experiments, *Ptf1a*^Cre/+^, *Kras*^LSLG12D/+^ *Trp53*^fl/fl^, and *Mir181ab1*^–/–^ mice were on a C57BL/6 background. Mice on a mixed background were backcrossed for at least 4 generations. Male and female mice were used indistinctively for mouse genetics experiments. Only *Rag2^–/–^* female mice were used for allograft experiments. Further details of mouse work can be found in the Supplemental Methods.

### MRI.

MRI experiments were performed on *Ptf1a*^Cre/+^ *Kras*^LSL-G12D/+^ *Trp53*^fl/fl^ and *Ptf1a*^Cre/+^ *Kras*^LSL-G12D/+^ *Trp53*^fl/fl^ *Mir181ab1*^fl/fl^ mutant mice at the age of 7 weeks. MRI was performed using the Biospec USR70/30 (Bruker Biospin MRI). MR images were analyzed using open-source Horos processing software. Tumor volume (*V*) was assessed, using 3D volumetric measurements according to the modified Simpson rule,

(Equation 1)
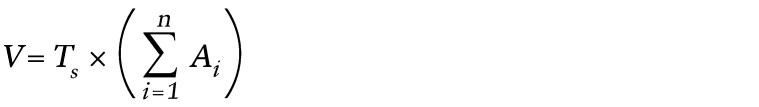


where *T_s_* is the thickness of each slice, *i* is the individual slice number, and *n* is the total number of slices.

### Tumor area analysis.

Micrographs of H&E-stained slides from multiple lung sections for each sample were obtained and pictures taken at ×20 magnification. Bioquant software was used to montage the entire lung sections and to calculate tumor area for each sample. Tumor area was calculated with the manual measurement feature in Bioquant.

### Histology and IHC.

IHC was performed on formalin-fixed, paraffin-embedded mouse and human tissue sections using a biotin-avidin method as described previously (81). The following antibodies were used: anti-CC3 (1:200), anti-Ki67 (1:1,000), and anti-MUC5AC (1:500). Sections were developed with DAB and counterstained with hematoxylin. Pictures were taken using a Leica microscope equipped with the LAX software. IHC analysis was performed using ImageJ software.

### qPCR analysis.

RNA was analyzed as previously described (82). MEFs were treated for 72 hours with adenoviruses and grown in fresh medium for an additional 72 hours. Then, MEFs were plated and RNA harvested after 24 hours with TRIzol reagent (Invitrogen). KLA cells were treated for 48 hours with adenoviruses and then fresh medium was added for 24 hours before RNA isolation. cDNA was synthesized with a DyNAmo cDNA synthesis kit (F470, New England Biolabs) and qRT-PCR was performed using SYBR Green (Applied Biosystems). *GAPDH* and *HPRT* were used as housekeeping genes. For miRNA analyses, TaqMan assays were used (miR181a, ID 000480; miR181b, ID 001098). *RNU6* and *Sno135* were reference genes in mouse cell lines. *RNU6* and *RNU48* were used as housekeeping genes in human cell lines.

### Northern blot.

The nonradioactive miRNA Northern Blot Assay Kit including 2 gels (NB-0001), miR181a-5p(HS) (HP-0081), and miR181b-5p(HS) (HP-0082) (Signosis) was used to study miR181a1 and miR181b1 expression in KLA mouse lung tumor cells following the instructions from the manufacturer. RNA (10 μg) from KLA cells treated with adEmpty or adCre for 48 hours was used. A ChemiDoc Gel Imaging System (Bio-Rad) was used to acquire Northern blot images.

### Cell proliferation assay.

Cell proliferation in 2D was assessed using the CellTiter 96 AQueous Non-Radioactive Cell Proliferation Assay, MTS (Promega). Experiments were read on the indicated days according to manufacturer’s instructions and normalized to day 1 after seeding. Cell proliferation in 3D was measured by the CellTiterGLO (Promega) and normalized to day 1 after seeding.

### Clonogenic assay.

Two thousand H1792 cells were seeded in a 6-well plate for 10 days. Then, wells were washed with Dulbecco’s PBS (DPBS) and fixed for 10–15 minutes with 4% formaldehyde. Fixed cells were dyed with 0.5% crystal violet for 5 minutes. Crystal violet was diluted in DPBS/10% acetic acid and absorbance was measured at 570 nm.

### CRISPR/Cas9 strategy.

sgRNAs complementary to the 5′ and 3′ flanks of mir181ab1 gDNA were designed using the crispr.mit.edu tool. Then, a combination of 5′–3′ guides was subcloned in pX333 from Addgene (83). Cells were transduced with a 10:1 ratio of pX333/pMax GFP (Lonza). Single GFP-positive cells were sorted in a p96 plate using a FACSAria IIU (Becton Dickinson) and expanded for experiments.

### Phospho–histone 3 immunofluorescence.

KLA wt/wt and KLA mut/mut adCre–treated (10,000 cells/well), KPC181^ko^ wt/wt and KPC181^ko^ mut/mut, H1792 control, and CRISPRed miR181 cells were seeded in an 8-chamber polystyrene vessel, tissue culture–treated glass slides (354108; Falcon) and cultured for 72 hours. Then, phospho–histone 3 (p-H3) immunofluorescence was performed using an anti–p-H3 (S10) antibody (1:800, 9701, Cell Signaling Technology) and Alexa Fluor 594–goat anti-rabbit antibody (A11037, Life Technologies). Nuclei were stained with DAPI (1:1000). Images were taken at ×5 and ×20 magnification using a fluorescence microscope (Axio Imager M1; Zeiss) and percentage of p-H3–positive cells was analyzed using Fiji software.

### Pharmacological inhibitor and TGF-β treatment.

For dasatinib (Selleck) treatment, KLA wt/wt and KLA mut/mut cells treated with adCre or H1792 control and CRISPR–knocked out cells were seeded in 96-well plates. After overnight culture, dasatinib was added at the indicated concentrations for 72 hours. For TGF-β treatment, KLA and KPC cells were seeded and treated for 3 hours with 10 ng/mL recombinant human TGF-β (Preprotech).

### RNA sequencing analysis.

Samples were prepared with the Illumina TruSeq Stranded mRNA kit as per the manufacturer’s indications and sequenced as reverse paired-end (100 bp) runs on the HiSeq 4000 sequencer. Details of RNA sequencing analysis are provided in Supplemental Methods. The RNA sequencing data have been deposited in NCBI’s Gene Expression Omnibus database (GEO accession number GSE128478).

### Pathway analysis.

The biological knowledge extraction and network representation was complemented through the use of IPA (Ingenuity Systems, Qiagen).

### Luciferase assay.

Complementary oligonucleotides containing the wild-type or a mutated sequence of the *Nexmif* 3′UTR were subcloned into the psiCHECK-2 vector using XhoI and NotI restriction sites. KLA wt/wt and mut/mut cells pretreated with adCre (48 hours) were seeded in a 24-well plate. Cells were transfected with 0.5 μg of plasmids using the X-tremeGENE HP DNA Transfection Reagent (6366244001, Roche) for 72 hours. Media were refreshed for 24 hours and Renilla and firefly luciferase activities were measured using the Dual Luciferase Report Assay System (Promega) following the manufacturers’ instructions and a plate reader (Berthold). Wild-type 3′UTR primers (5′–3′): Forward aagttctcgaggctgcctacagagttttgaatgtacttactagactttagttagagaccctttttatgaatgtaacctgtttctgtttgtttaaatatttgtgactgaatgtatggtgaaactgtcatgcggccgcttgaa; Reverse ttcaagcggccgcatgacagtttcaccatacattcagtcacaaatatttaaacaaacagaaacaggttacattcataaaaagggtctctaactaaagtctagtaagtacattcaaaactctgtaggcagcctcgagaactt. Mutated 3′UTR primers (5′–3′): Forward aagttctcgaggctgcctacagagtttggggggacttactagactttagttagagaccctttttaggggggaacctgtttctgtttgtttaaatatttgtgacggggggatggtgaaactgtcatgcggccgcttgaa; Reverse ttcaagcggccgcatgacagtttcaccatccccccgtcacaaatatttaaacaaacagaaacaggttcccccctaaaaagggtctctaactaaagtctagtaagtccccccaaactctgtaggcagcctcgagaactt.

### Nexmif overexpression.

The Nexmif ORF (NM_001077354.2) was used to replace Nanog in the pSIN-EF2-Nanog-Puro vector (https://www.addgene.org/16578/) using SpeI and EcoRI restriction sites. Lentiviruses were produced in HEK293T cells. KLA cells were infected and selected with 5 μg/mL puromycin.

### Survival analysis.

Survival analysis was conducted on the selected gene set using RNA sequencing data sets of LUAD patients (TCGA) and PDAC (ICGC) (3). The log-rank test was used to calculate the statistical significance of differences observed among Kaplan-Meier curves, as previously described (84).

### Statistics.

Sample size was chosen using http://www.biomath.info/power/ttest.htm or based on similar experiments previously published by the authors. For comparison of 2 groups, samples were explored for normality (Shapiro-Wilk test) and variance (Levene test). Groups with normal distribution of samples were analyzed with a *t* test. Non-normal samples were analyzed using a Mann-Whitney test (equal variances) or a median test (unequal variances). For comparison of more than 2 groups, a residual test was performed to study normality and the Levene test assessed homoscedasticity. ANOVA, Brown-Forsythe, Kruskal-Wallis, or a median test was performed depending on data distribution. A Dunnett’s post hoc test explored paired comparisons. All analyses were 2-tailed. Error bars correspond to either standard deviation (SD, *n* < 8) or standard error of the mean (SEM, *n* ≥ 8) for parametric variables, and interquartile range for nonparametric variables, as indicated for each experiment. Statistical analyses were done using SPSS software.

### Study approval.

All experiments in mice were performed according to the MD Anderson Cancer Center Institutional Animal Care and Use Committee (IACUC, protocol 00001636), UCSF Committee on Animal Care (APLAC), and the University of Navarra Ethical Committee on Animal Research (CEEA, protocol 068-13). Regarding human data, only normalized/processed data of coded clinical information were made available to this study to preserve patients’ anonymity.

## Author contributions

EASC and SV conceived the project. CZC, PKM, EASC, and SV designed and planned the experiments. PKM, EASC, and SV supervised the work. EG and AGL carried out computational analyses. KV, OE, KK, SH, AT, LCS, NMF, RF, TQS, AV, MR, REC, IM, NR, PF, FL, JL, and MPS contributed to experimental design and execution. CZC provided *Mir181*^–/–^ and *Mir181*^fl/fl^ mice. KV, OE, KK, SH, CZC, PKM, EASC, and SV were responsible for the data analysis and interpretation. KV, OE, PKM, EASC, and SV wrote the manuscript and were in charge of the manuscript preparation. All the authors reviewed and edited the manuscript.

## Supplementary Material

Supplemental data

## Version 1 12/24/2019

In-Press Preview

## Version 2 03/09/2020

Electronic publication

## Version 3 04/01/2020

Print issue publication
