# Supplementary material for: The Mir181ab1 cluster promotes KRAS-driven oncogenesis and progression in lung and pancreas
Source: J Clin Invest. 2020 Mar 9;130(4):1879–95. doi: 10.1172/JCI129012 (PMC7108928; doi:10.1172/JCI129012)
Supplement: Supplemental data [file jci-130-129012-s298.pdf]

THE MIR181AB1 CLUSTER PROMOTES KRAS-DRIVEN ONCOGENESIS AND PROGRESSION IN LUNG AND PANCREAS

Karmele Valencia, Oihane Erice, Kaja Kostyrko, Simone Hausmann, Elizabeth Guruceaga, Anuradha Tathireddy, Natasha M. Flores, Leanne C. Sayles, Alex G. Lee, Rita Fragoso, Tian-Qiang Sun, Adrian Vallejo, Marta Roman, Rodrigo Entrialgo-Cadierno, Itziar Migueliz, Nerea Razquin, Puri Fortes, Fernando Lecanda, Jun Lu, Mariano Ponz-Sarvise, Chang-Zheng Chen, Pawel K. Mazur, E. Alejandro Sweet-Cordero, Silvestre Vicent

SUPLEMENTARY FIGURES AND LEGENDS

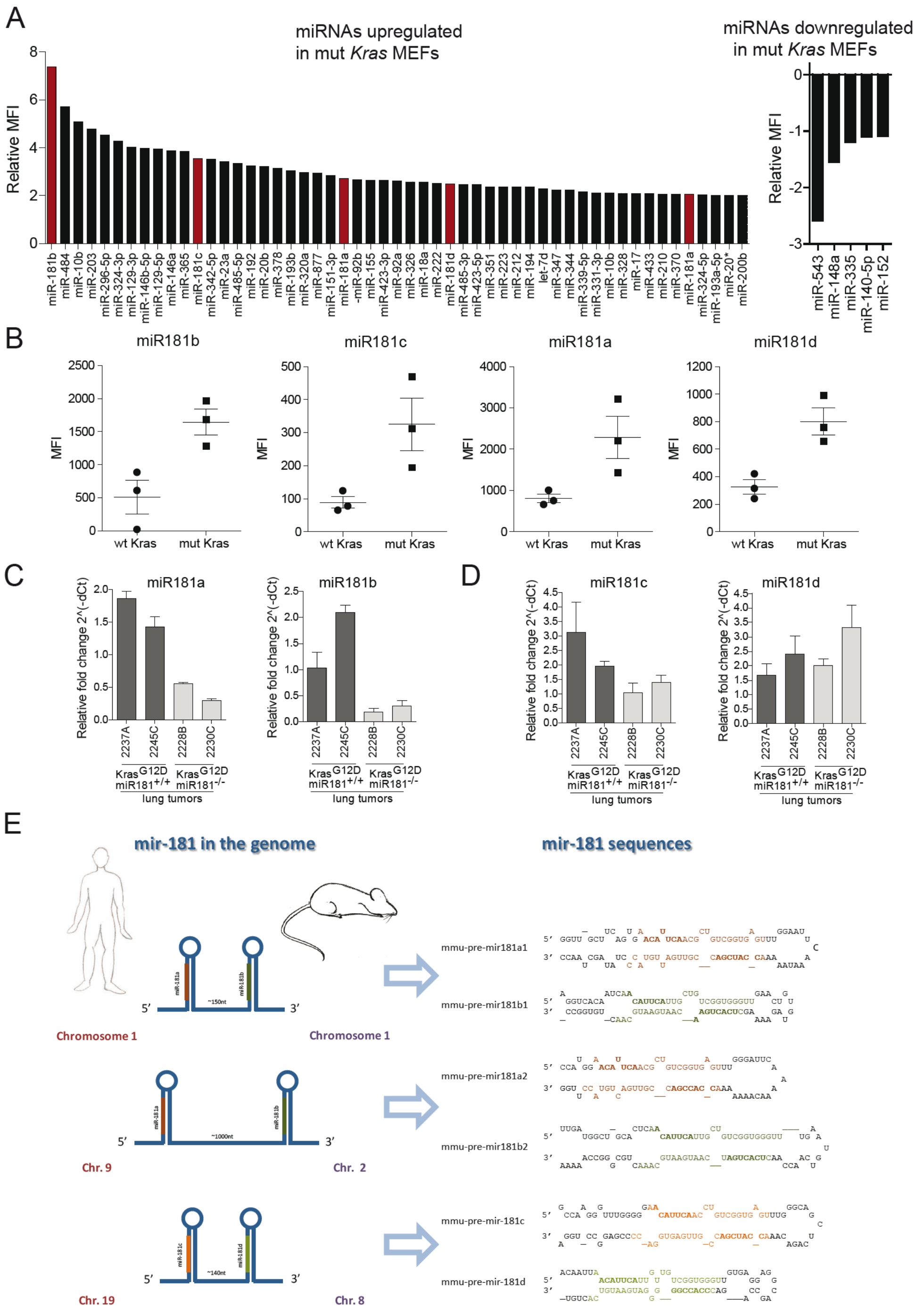

Suppl. Figure 1 (related to Figure 1). (A) List of upregulated (left) and downregulated (right) miRNAs upon mutant *Kras* activation in mouse embryo fibroblasts (MEFs) assessed by luminex. Relative MFI (mean fluorescence intensity) is normalized to expression of miRNAs in wild-type MEFs. (B) Absolute MFI expression of the miR181 family members in wild-type and mutant *Kras* MEFs (n=3). (C) Expression of miR181a and miR181b in $K181^{+/+}$ and $K181^{-/-}$ lung tumors. (D) Expression of miR181c and miR181d in $K181^{+/+}$ and $K181^{-/-}$ in the same lung tumors as in C. (E) Schematic representation of the three miRNA clusters of the miR181 family in mouse and human (left). Pre-miRNA sequences of mouse miR181 clusters (right). Nucleotide sequence in bold correspond to the seed sequences of the mature miR181 members.

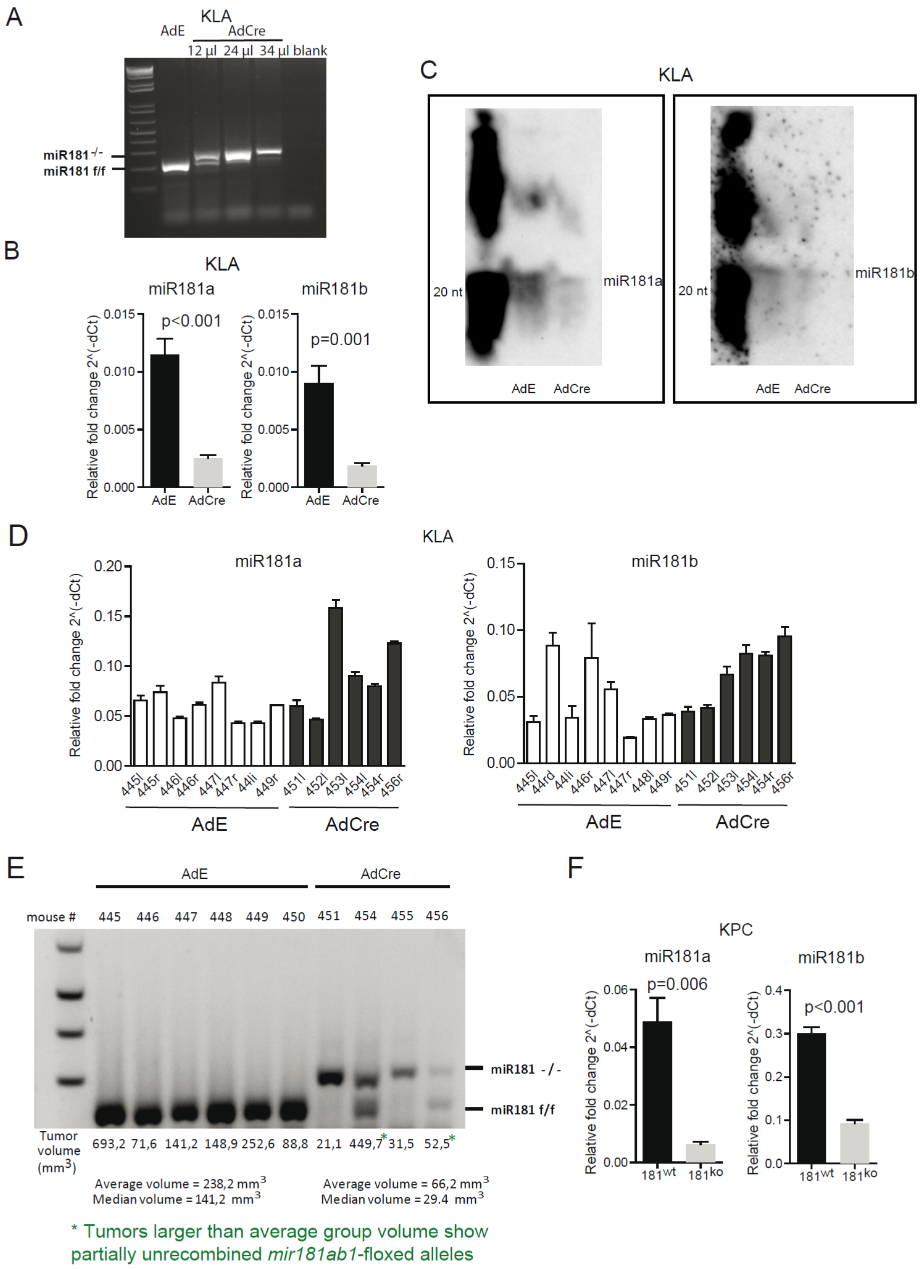

Suppl. Figure 2 (related to Figure 5). (A) Genotyping PCR showing the conditions for recombination of the *mir181ab1* floxed alleles in KLA cells. (B) MiR181a and miR181b expression in mouse lung adenocarcinoma *Kras* mutated cells (KLA) treated for 48 h with cre recombinase (adCre) or empty (adE) adenovirus (n=3) compared by *t*-test. (C) Northern blot analysis of RNA samples from the same cells as in B. (D) Quantitative PCR of miR181a and miR181b expression in tumors. (E) Genotyping PCR showing a loss of recombination in tumors from KLA adCre. (F) miR181a and miR181b expression in mouse pancreatic adenocarcinoma (KPC) *Mir181ab1*$^{+/+}$ and *Mir181ab1*$^{f/f}$ cells (n=3) compared by *t*-test.

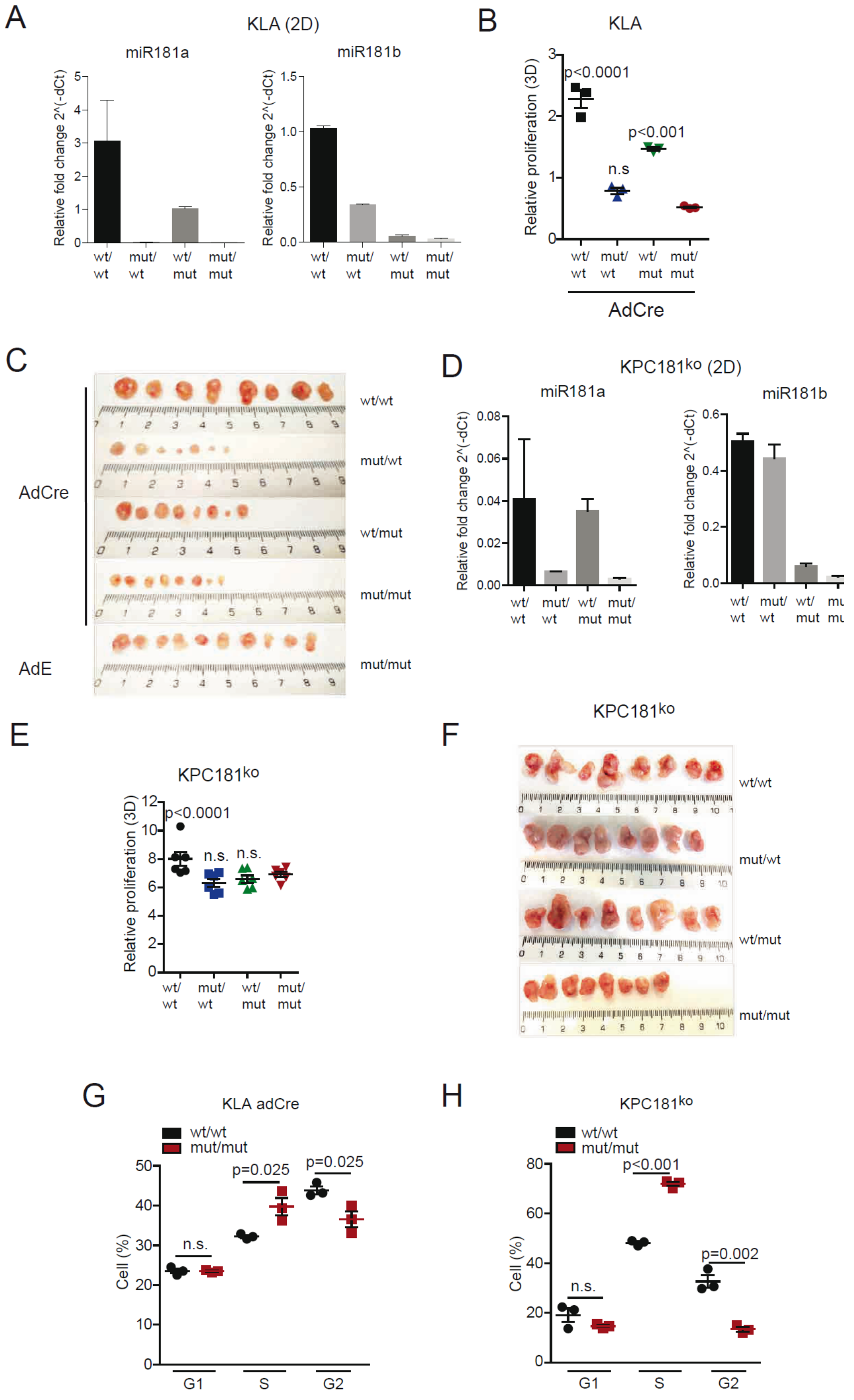

Suppl. Figure 3 (related to Figure 6). (A) Quantitative PCR of miR181a and miR181b expression in KLA cells overexpressing exogenous *mir181a1* and *Mir181b1* (wt/wt), *Mir181a1* (wt/mut), *Mir181b1* (mut/wt) or a control sequence (mut/mut) assessed previously to functional assays (n=3). (B) Relative proliferation of KLA organoids transduced with the different miR181 constructs measured by CellTiterGLO at day 3 (n=3) compared by ANOVA. Results are the average of three independent experiments. (C) Tumor images of the different groups described. (D) Quantitative PCR of miR181a and miR181b expression in miR181^{ko} pancreatic cancer cells

overexpressing exogenous *Mir181a1* and *Mir181b1* (wt/wt), *Mir181a1* (wt/mut), *Mir181b1* (mut/wt) or a control sequence (mut/mut) assessed previously to functional assays (n=3). (E) Relative proliferation of miR181^{ko} pancreatic cancer organoids transduced with the different miR181 constructs measured by CellTiterGLO at day 3 (n=6) compared by ANOVA. (F) Tumor images of the different groups described. (G) Cell cycle distribution assessed with EdU and 7AAD in KLA wt/wt adCre and KLA mut/mut adCre cells 24 h after starving synchronization (n=3). The percentage of cells in each phase is compared by *t*-test. Cell cycle assays are representative of three independent experiments. (H) Cell cycle distribution assessed with EdU and 7AAD in KPC181^{ko} wt/wt and mut/mut cells 14h after starving synchronization (n=3). Results are representative of three independent experiments and were compared by *t*-test.

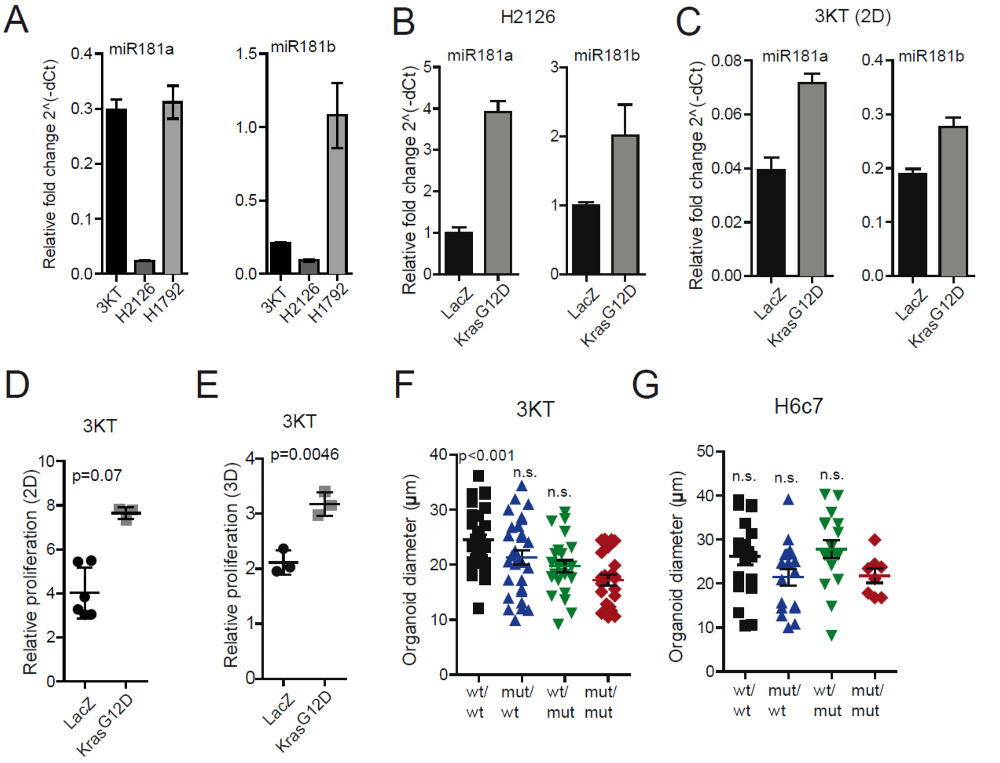

Suppl. Figure 4 (related to Figure 7). (A) MiR181a and miR181b expression in human immortalized normal bronchio-epithelial cells (3KT), wild-type (H2126) and mutant (H1792) *KRAS* lung cancer cells (n=3). (B) H2126 expressing control (LacZ) and mutated *KRAS* (G12D) expressing vectors (n=3). (C) MiR181a and miR181b expression in normal human bronchial epithelial immortalized cells 3KT transduced with control (LacZ)- and *KRAS* mutated (G12D)-expressing vectors (n=3). (D) Cell proliferation of 3KT cells expressing control (lacZ) and mutant *KRAS* (G12D) expressing vectors assessed by MTS (n=6) and compared by *t*-test. (E) Relative proliferation of organoids from 3KT control and overexpressing *KRAS*^{G12D} measured at day 3 by CellTiterGLO (n=3) and compared by *t*-test. (F and G) Quantification of organoid size from 3KT (F) and H6c7 (G) cells expressing the different *Mir181a1* and *Mir181b1* constructs (n=8-35). Results were compared by ANOVA.

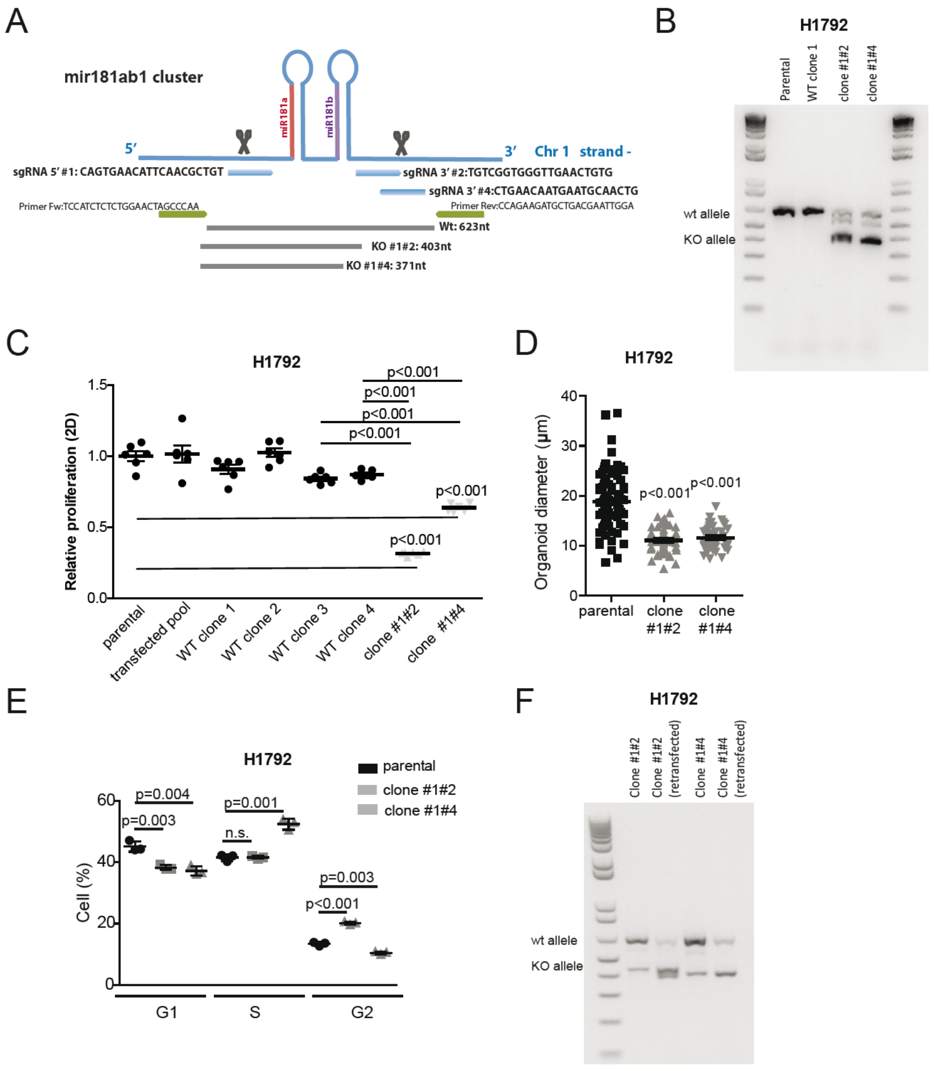

Suppl. Figure 5 (related to Figure 8). (A) Schematic representation of the CRISPR/Cas9 strategy designed to knockout the miR181ab1 cluster from human lung cancer cells expressing mutant *KRAS*. (B) Genotyping PCR of H1792 parental cells, a mir181ab1 wild-type clone and two mir181ab1-CRISPR'ed clones. (C) Cell proliferation analysis assessed by MTS in parental, sgRNA transfected pool cells, wild-type miR181ab1 (WT) and partially miR181ab1-knockedout (KO) cell clones (n=6). Results were compared by ANOVA. (D) Quantification of organoid diameter at day 3 post-seeding in 3D cultures of H1792 mir181ab1-CRISPR'ed cells (n=46-90). Results were compared by ANOVA. (E) Cell cycle distribution assessed with EdU and 7AAD in synchronized H1792 control and mir181ab1-CRISPR'ed clones 24h after seeding (n=3). Results were compared by *t*-test. (F) Genotyping PCR of H1792 CRISPR'ed clones re-transfected with the same sgRNAs.

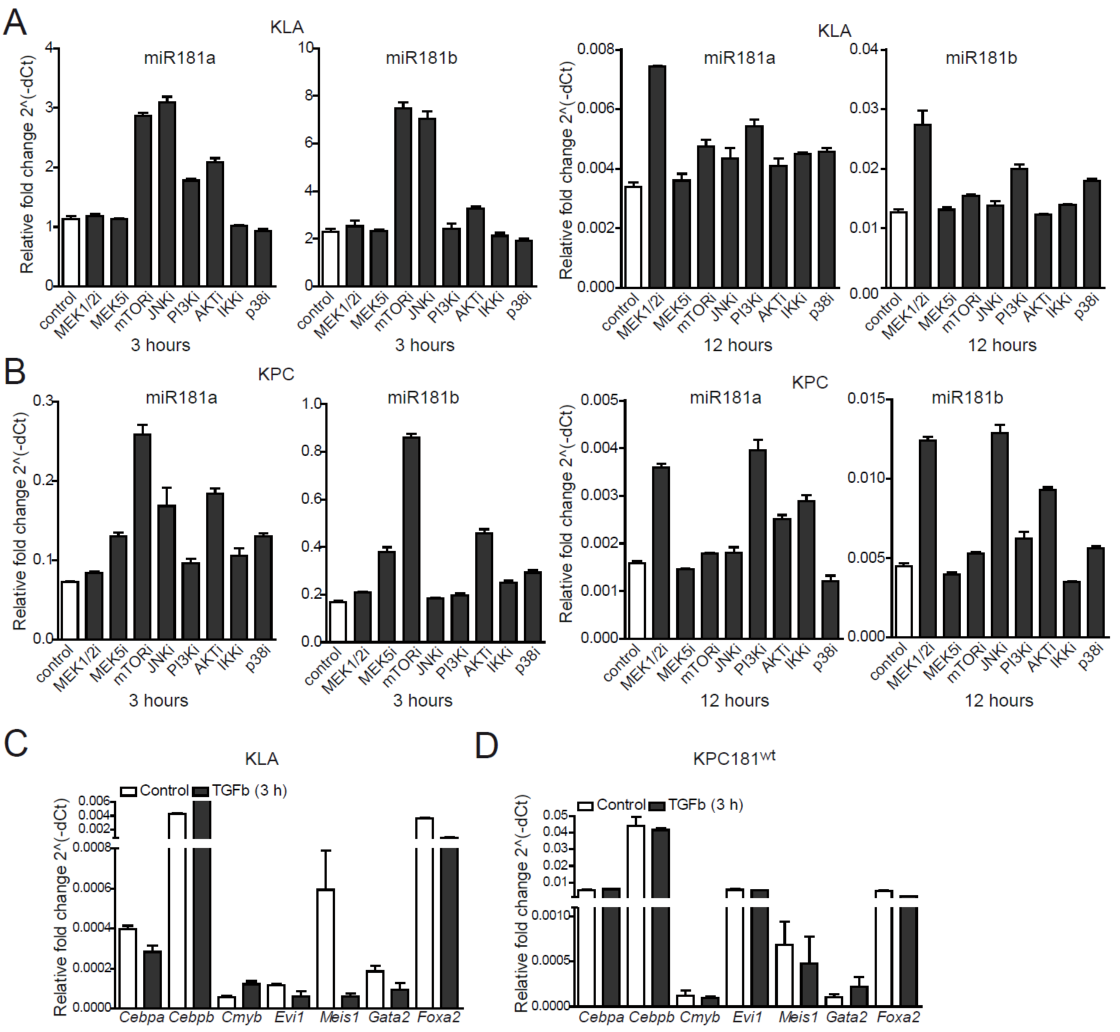

Suppl. Figure 6 (related to Figure 9). (A and B) MiR181a and miR181b expression in mouse lung cancer (KLA) and pancreatic cancer (KPC) cells treated with pharmacological inhibitors to the indicated kinases for 3 and 12 h (n=3). MEK1/2i: trametinib (1 µM), BIX02189 (MEK5i, 10 µM), SP600125 (JNKi, 20 µM), GSK2126458 (Pi3Ki, 0.1 µM), SB203580 (p38i, 20 µM), IKK-16 (IKKi, 1 µM), and LY294002 (AKTi, 10 µM). (C and D) Quantitative PCR to assess the expression of the indicated transcription factors (TFs) in mouse lung cancer (KLA) and pancreatic cancer (KPC) cells. (n=3). Results were compared by *t*-test. Error bars represent SD.

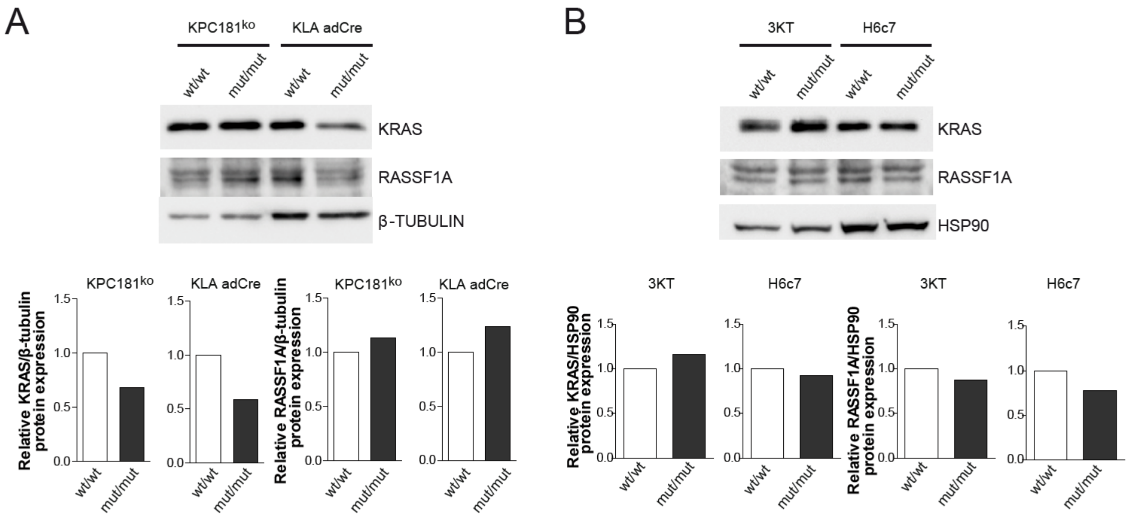

Suppl. Figure 7 (related to Figure 10). (A) Representative images by Western blot and densitometry analysis of KRAS and RASSF1A protein expression in miR181ab1-deficient and proficient mouse lung cancer (KLA) and pancreatic cancer (KPC) cells. (B) Representative images by Western blot and densitometry analysis of KRAS and RASSF1A protein expression in human lung (3KT) and pancreas (H6c7) immortalized epithelial cells expressing a control vector (mut/mut) and miR181ab1 (wt/wt). Data are representative of three independent experiments.

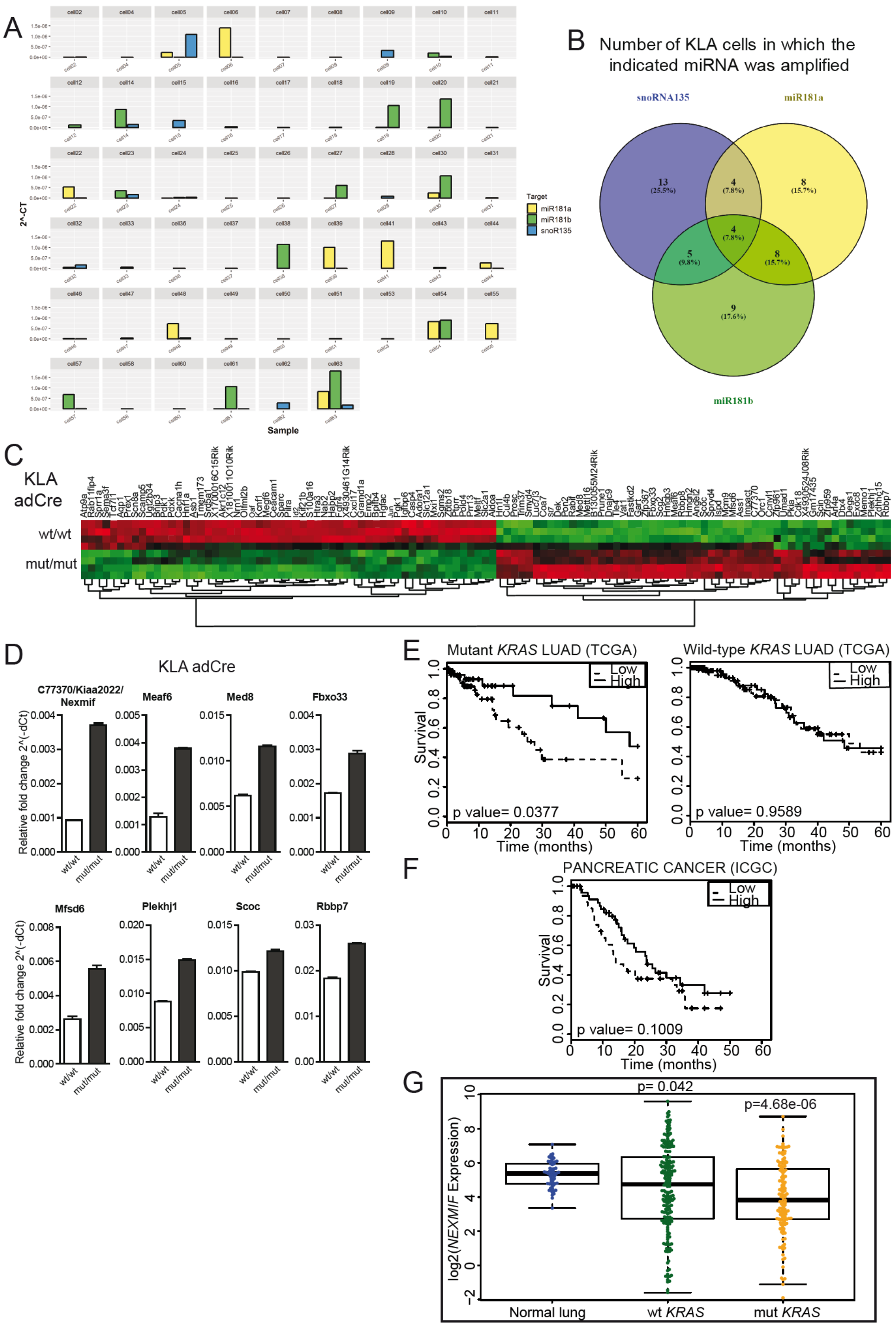

Suppl. Figure 8 (related to Figure 10). (A) Fluidigm analysis of single-cell miRNA expression in random cells from a population of mouse Kras-driven lung cancer cells (KLA). (B) Venn diagram showing distribution of miR181a, miR181b and snoR135 in the sampled cells analyzed in A. (C) Heat map of differentially expressed genes between KLA wt/wt and mut/mut adCre-treated cells. 54 downregulated (green) and 57 upregulated (red) genes were obtained (B>0 and logFC/0.5/). (D) Quantitative PCR to quantify mRNA of indicated genes in KLA wt/wt and mut/mut adCre-treated cells. (E) Kaplan–Meier plot of lung adenocarcinoma (LUAD) patients from TCGA stratified

based on the median expression of *NEXMIF* (log-rank test). (F) Kaplan–Meier plot of pancreatic ductal adenocarcinoma (PDAC) patients from ICGC based on the median expression of *NEXMIF* (log-rank test). (G) NEXMIF expression levels (log2) in normal lung, wild-type *KRAS* (p=0.042, FDR=0.0553) and mutant *KRAS* (p=p=4.68e-06, FDR=9.79e−06) LUAD patients (Wilcoxon test).

UNCROPPED WESTERN BLOTS

Western blot (whole membrane images) for Suppl. Figure 7A

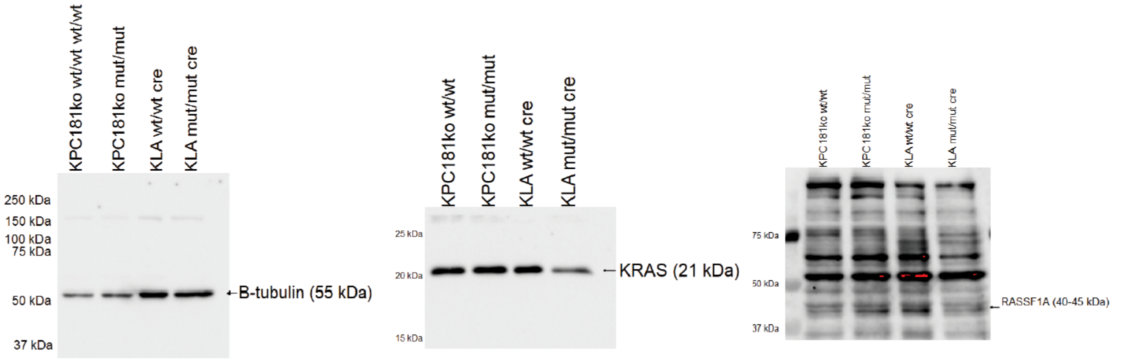

Western blot (whole membrane images) for Suppl. Figure 7B

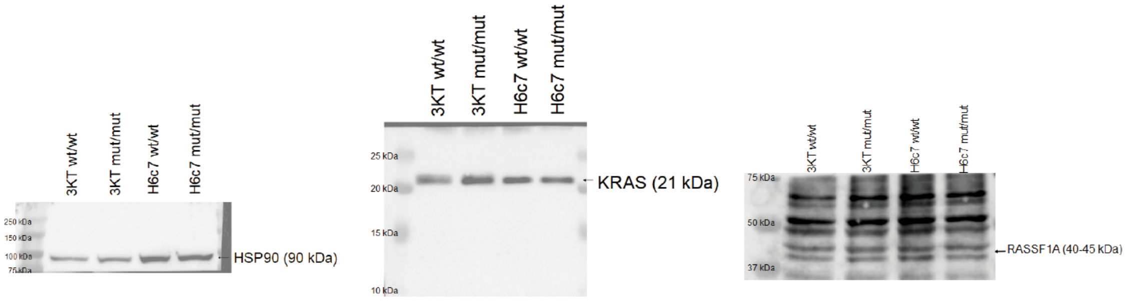

SUPPLEMENTARY TABLES

gene	logFC	FC	AveExpr	t	P.Value	adj.P.Val	B
Ispd	-3,10146	0,116511	-0,41965	-4,80655	0,000208	0,047047	0,853552
X4930524.	-2,79529	0,144057	-0,27335	-4,582	0,000328	0,049739	0,440899
Scg2	-2,67912	0,156136	1,585956	-5,6484	4,01E-05	0,030092	2,339631
Scin	-2,47859	0,17942	0,025837	-4,43311	0,000444	0,052313	0,164182
C77370	-2,37216	0,193156	4,683384	-4,44096	0,000437	0,052313	0,178832
Zdhhc15	-2,29238	0,204139	-0,04238	-5,28315	8,11E-05	0,038922	1,707624
Orc1	-1,91304	0,265533	3,710747	-5,02922	0,000134	0,042177	1,256488
Pkia	-1,64571	0,319589	1,259976	-6,19879	1,44E-05	0,017246	3,251729
Meaf6	-1,55303	0,340793	3,748044	-4,41107	0,000464	0,053215	0,123034
Mettl16	-1,52936	0,346432	5,290169	-6,05216	1,88E-05	0,020096	3,013591
B130055M	-1,47699	0,359236	0,533247	-5,06922	0,000123	0,042177	1,328153
Gm17435	-1,40986	0,376347	0,086544	-4,5971	0,000318	0,049739	0,468834
Zfp959	-1,29776	0,406756	2,324033	-4,54654	0,000352	0,049739	0,375221
Dek	-1,28982	0,409001	7,404103	-4,97334	0,000149	0,042343	1,155982
Hmgn2	-1,25007	0,420428	6,597047	-5,03284	0,000133	0,042177	1,262976
Coa7	-1,23417	0,425087	3,266092	-4,74294	0,000237	0,047313	0,737272
Ass1	-1,22527	0,427717	3,765744	-4,84434	0,000193	0,047047	0,922385
Impact	-1,21794	0,429895	5,0545	-4,61101	0,000309	0,049739	0,494552
Mcm9	-1,10185	0,465919	2,986358	-4,97561	0,000149	0,042343	1,160079
Zfp367	-1,02719	0,490664	5,675561	-4,48208	0,000401	0,052313	0,255448
St7	-1,02585	0,49112	4,075827	-4,54645	0,000352	0,049739	0,375046
Dnajc9	-1,00372	0,498711	6,504155	-4,55952	0,000343	0,049739	0,399272
Fbxo33	-0,99743	0,500893	4,830301	-4,97881	0,000148	0,042343	1,165846
Hmgb3	-0,94431	0,519679	5,912464	-4,53033	0,000364	0,050431	0,345134
Zfp961	-0,91076	0,531903	3,660411	-4,9228	0,000165	0,043152	1,064722
Vat1	-0,9071	0,533255	7,80361	-6,35087	1,09E-05	0,016351	3,494926
Tle4	-0,9015	0,535331	5,024941	-5,30365	7,79E-05	0,038922	1,743643
Mfsd6	-0,89103	0,539228	5,100302	-4,92156	0,000165	0,043152	1,062492
Fastkd2	-0,88615	0,541054	5,3302	-5,61051	4,31E-05	0,030442	2,275033
Plekhj1	-0,87504	0,545241	5,025659	-4,54603	0,000352	0,049739	0,374266
Rbbp8	-0,87219	0,546317	4,996987	-4,40833	0,000467	0,053215	0,117907
Trim37	-0,85708	0,552068	5,353267	-5,17456	0,0001	0,039882	1,515839
Prune1	-0,83134	0,562009	6,132979	-4,81053	0,000207	0,047047	0,860804
Angel2	-0,82697	0,563713	5,583424	-4,77634	0,000221	0,047047	0,798383
Ccnyl1	-0,82008	0,566411	5,322535	-4,38794	0,000487	0,053546	0,079795
Exoc8	-0,81528	0,568297	4,710066	-4,39015	0,000484	0,053546	0,083933
Med8	-0,80048	0,574157	4,928365	-4,66307	0,000278	0,049739	0,590577
Arl4a	-0,79701	0,575542	5,026207	-4,52017	0,000371	0,050637	0,326275
Luc7l3	-0,79307	0,577115	6,57277	-4,41158	0,000464	0,053215	0,123985
Smyd4	-0,73389	0,60128	3,801294	-4,45035	0,000428	0,052313	0,196341
Prosc	-0,72132	0,606543	5,546202	-4,82995	0,000199	0,047047	0,896192
Degs1	-0,70931	0,611612	6,764142	-4,46359	0,000417	0,052313	0,221016
Hn1l	-0,70655	0,612786	6,999164	-5,12533	0,000111	0,040172	1,428321
Scoc	-0,70106	0,615121	5,12228	-4,60638	0,000312	0,049739	0,485992
Memo1	-0,70021	0,615483	6,053818	-5,00551	0,00014	0,042343	1,213886
Pon2	-0,68483	0,622081	6,377909	-4,52779	0,000366	0,050431	0,340431

Rbbp7	-0,67993	0,624194	8,025247	-4,44656	0,000432	0,052313	0,189287
Umad1	-0,66965	0,628661	3,325217	-4,64605	0,000288	0,049739	0,559205
Srpyd4	-0,65892	0,633351	4,171991	-5,45977	5,76E-05	0,033015	2,015807
Dtx4	-0,65304	0,635939	5,583794	-4,36237	0,000513	0,055408	0,031941
Cdk18	-0,64167	0,640971	7,655402	-4,62489	0,0003	0,049739	0,520179
Rabif	-0,63058	0,645915	5,590508	-4,69256	0,000262	0,049739	0,644825
Gart	-0,62185	0,649839	7,10351	-4,43774	0,000439	0,052313	0,172827
Cul4b	-0,57708	0,670321	5,628627	-4,46174	0,000418	0,052313	0,217582
Scn8a	0,606456	1,522514	6,351055	4,770046	0,000224	0,047047	0,786878
Gramd1a	0,63886	1,557098	4,868252	4,354468	0,000521	0,05581	0,017133
Prr13	0,776539	1,713016	7,687826	4,573604	0,000333	0,049739	0,425366
Pgk1	0,786319	1,724668	9,79188	4,694852	0,000261	0,049739	0,649034
Mxi1	0,789816	1,728854	6,609015	4,670091	0,000274	0,049739	0,603494
Hgfac	0,822607	1,768598	6,566403	5,467961	5,67E-05	0,033015	2,029986
Emp2	0,824061	1,770382	6,249991	5,913054	2,44E-05	0,020884	2,784397
Ugt2b34	0,83256	1,780842	6,442683	5,195765	9,62E-05	0,039882	1,553424
Aldoa	0,847573	1,799471	10,79633	4,404618	0,00047	0,053215	0,11098
Sprr1a	0,895205	1,859874	8,928798	4,797242	0,000212	0,047047	0,836562
Asb1	0,908244	1,87676	4,877766	6,000144	2,07E-05	0,020096	2,928263
Pilra	0,915094	1,885692	5,098882	5,433881	6,06E-05	0,033015	1,970932
Slc2a1	0,927149	1,901514	8,08264	5,436644	6,02E-05	0,033015	1,975727
Zbtb18	0,954668	1,938134	6,742048	4,947963	0,000157	0,042803	1,110205
Sgms2	0,96712	1,954934	6,61472	4,569046	0,000336	0,049739	0,416925
Pold4	1,002802	2,003888	5,251565	4,456156	0,000423	0,052313	0,20717
Kcnf1	1,018149	2,025318	3,911607	5,179162	9,94E-05	0,039882	1,523999
Akr1c12	1,02429	2,033959	6,223321	4,964953	0,000152	0,042343	1,140865
Oaf	1,044766	2,063032	5,60039	4,437007	0,00044	0,052313	0,171461
Hnf1a	1,051553	2,07276	4,022211	4,497869	0,000389	0,051812	0,28483
Tcf7l1	1,078937	2,112479	2,655266	6,480931	8,63E-06	0,014791	3,699834
Rab11fip4	1,099549	2,142877	1,449759	4,745793	0,000235	0,047313	0,742492
Nab2	1,120337	2,173977	4,094699	4,856319	0,000189	0,047047	0,944171
Srd5a1	1,189146	2,280177	6,537646	4,627858	0,000299	0,049739	0,525656
Megf6	1,213756	2,319407	4,262092	4,622675	0,000302	0,049739	0,51609
S100a16	1,226401	2,339825	5,71018	6,510321	8,19E-06	0,014791	3,745747
Pdxk	1,244048	2,368622	5,972191	5,33091	7,39E-05	0,03855	1,791427
Sema3f	1,257104	2,390155	1,2939	4,610349	0,000309	0,049739	0,493326
Ptprr	1,355383	2,558651	4,519278	4,436271	0,000441	0,052313	0,170087
Olfml2b	1,389588	2,620038	4,257446	5,57626	4,61E-05	0,030694	2,216447
Tmem173	1,412653	2,662262	3,209941	4,431792	0,000445	0,052313	0,16173
X1700016	1,414851	2,666322	5,495634	4,393369	0,000481	0,053546	0,08995
Sparc	1,442207	2,717363	4,418904	6,859513	4,43E-06	0,010622	4,280165
Pdk1	1,465118	2,76086	7,048361	4,762603	0,000227	0,047047	0,773265
X1810011	1,478667	2,78691	6,016246	4,800241	0,000211	0,047047	0,842036
Scamp5	1,494028	2,816743	4,073673	4,505942	0,000382	0,05154	0,299841
Atp9a	1,6875	3,22098	2,698747	7,360225	1,89E-06	0,005658	5,011047
Habp2	1,688717	3,223698	4,737472	4,37871	0,000496	0,054072	0,062526
Aqp1	1,800169	3,48261	5,952625	11,4036	5,75E-09	6,89E-05	9,561512

Id2	1,826761	3,547397	7,271218	5,0503	0,000128	0,042177	1,294284
Prex1	1,869828	3,654891	3,023384	7,914123	7,63E-07	0,003052	5,771985
Fgfr4	1,933544	3,819923	1,974413	4,836887	0,000196	0,047047	0,908824
Casp4	1,948298	3,859189	0,293205	4,598594	0,000317	0,049739	0,471602
Jun	1,984095	3,956144	6,306516	5,228992	9,02E-05	0,039882	1,61219
Ceacam1	2,096591	4,276975	4,673001	8,919302	1,63E-07	0,000977	7,031675
Bnip3	2,09761	4,279997	6,22594	4,565669	0,000339	0,049739	0,410672
Cxcl17	2,24996	4,756697	1,877485	4,595841	0,000319	0,049739	0,466512
Nrn1	2,297109	4,91472	4,72136	4,577876	0,00033	0,049739	0,433276
Igfbp6	2,380637	5,207665	0,290894	5,165655	0,000102	0,039882	1,500031
Slc12a1	2,413917	5,329192	0,539988	6,221708	1,38E-05	0,017246	3,288619
Kif21b	2,455724	5,485884	4,006403	4,632433	0,000296	0,049739	0,534098
Bpifb4	2,510285	5,697327	3,4668	5,850447	2,74E-05	0,021915	2,680208
Htra3	2,517527	5,725996	2,817592	5,158651	0,000104	0,039882	1,487594
Cacna1h	3,174446	9,02825	2,545854	5,070118	0,000123	0,042177	1,329765
Meltf	3,356229	10,24061	0,811115	5,144642	0,000106	0,039882	1,462693
Adora1	3,545069	11,67272	0,445084	4,781687	0,000219	0,047047	0,808156
X4930461	3,653351	12,58253	0,353665	4,672264	0,000273	0,049739	0,607494

Suppl. Table 1 (related to Figure 10). Differentially expressed genes in KLA cells (B>0, logFC 0.5)

Collection(s):	MIR				
# overlaps shown:	10				
# genesets in collections:	221				
# genes in comparison (n):	43				
# genes in universe (N):	45956				
**Gene Set Name**	**# Genes in Gene Set (K)**	**# Genes in Overlap (k)**	**k/K**	**p-value**	**FDR q-value**
TGAATGT_MIR181A_MIR181B_MIR181C_MIR181D	484	8	0,0165	1,50E-08	3,31E-06
AGCATTA_MIR155	134	4	0,0299	7,81E-06	6,96E-04
GTGCCAA_MIR96	303	5	0,0165	9,45E-06	6,96E-04
CAGTATT_MIR200B_MIR200C_MIR429	469	5	0,0107	7,58E-05	4,19E-03
AACATTC_MIR4093P	142	3	0,0211	3,26E-04	1,44E-02
ACTGTGA_MIR27A_MIR27B	474	4	0,0084	1,00E-03	3,69E-02
CCCACAT_MIR2993P	54	2	0,037	1,19E-03	3,75E-02
CACTTTG_MIR520G_MIR520H	237	3	0,0127	1,44E-03	3,79E-02
ATGTTAA_MIR302C	243	3	0,0123	1,54E-03	3,79E-02
CAAGGAT_MIR362	66	2	0,0303	1,77E-03	3,90E-02
v7.1 miRBase					

Suppl. Table 2 (related to Figure 10). MSigDB analysis for miRNAs predicted to bind the seed sequence of genes in the downregulated list obtained from *Mir181ab1*-knockout cells reconstituted with exogenous expression of miR181ab1.

Gene	Associated.G	miRNA	name	pred_DBs	val_DBs	TsCons	TSNCons	MicroCosm	miRanda_sc	miRanda_s	miRanda_c	miRanda	PicTar_mcf	PicTar_mc	PicTar_m	PITA	Seed	miRTarbase	miRecords	Tarbase
ENSG000	ANGEL2	MIMAT0000256	hsa-miR-181a-5p	1	0	0	0	0	0	0	1	0	0	0	0	0	0	0	0	0
ENSG000	ANGEL2	MIMAT0000257	hsa-miR-181b-5p	1	0	0	0	0	0	0	1	0	0	0	0	0	0	0	0	0
ENSG000	ARL4A	MIMAT0000270	hsa-miR-181a-3p	2	0	0	0	0	0	1	0	0	0	0	0	0	1	0	0	0
ENSG000	ARL4A	MIMAT0000256	hsa-miR-181a-5p	1	0	0	0	0	0	0	1	0	0	0	0	0	0	0	0	0
ENSG000	ARL4A	MIMAT0000257	hsa-miR-181b-5p	1	0	0	0	0	0	0	1	0	0	0	0	0	0	0	0	0
ENSG000	CCNYL1	MIMAT0000256	hsa-miR-181a-5p	1	0	0	0	0	0	0	0	0	0	0	0	0	1	0	0	0
ENSG000	CCNYL1	MIMAT0022692	hsa-miR-181b-3p	1	0	0	0	0	0	0	0	0	0	0	0	0	1	0	0	0
ENSG000	CCNYL1	MIMAT0000257	hsa-miR-181b-5p	1	0	0	0	0	0	0	0	0	0	0	0	0	1	0	0	0
ENSG000	CUL4B	MIMAT0000256	hsa-miR-181a-5p	2	0	0	0	0	1	0	1	0	0	0	0	0	0	0	0	0
ENSG000	CUL4B	MIMAT0000257	hsa-miR-181b-5p	2	0	0	0	0	1	0	1	0	0	0	0	0	0	0	0	0
ENSG000	DEK	MIMAT0000256	hsa-miR-181a-5p	**4**	0	0	0	0	1	0	1	0	0	0	1	0	1	0	0	0
ENSG000	DEK	MIMAT0022692	hsa-miR-181b-3p	1	0	0	0	0	0	0	0	0	0	0	0	0	1	0	0	0
ENSG000	DEK	MIMAT0000257	hsa-miR-181b-5p	**4**	0	0	0	0	1	0	1	0	0	0	1	0	1	0	0	0
ENSG000	DTX4	MIMAT0000256	hsa-miR-181a-5p	**4**	0	0	0	0	0	0	1	0	0	0	1	1	1	0	0	0
ENSG000	DTX4	MIMAT0022692	hsa-miR-181b-3p	1	0	0	0	0	0	0	0	0	0	0	0	0	1	0	0	0
ENSG000	DTX4	MIMAT0000257	hsa-miR-181b-5p	**4**	0	0	0	0	0	0	1	0	0	0	1	1	1	0	0	0
ENSG000	EXOC8	MIMAT0000256	hsa-miR-181a-5p	2	0	0	0	0	1	0	0	0	0	0	0	0	1	0	0	0
ENSG000	EXOC8	MIMAT0000257	hsa-miR-181b-5p	2	0	0	0	0	1	0	0	0	0	0	0	0	1	0	0	0
ENSG000	FASTKD2	MIMAT0000256	hsa-miR-181a-5p	1	0	0	0	0	0	0	1	0	0	0	0	0	0	0	0	0
ENSG000	FASTKD2	MIMAT0000257	hsa-miR-181b-5p	1	0	0	0	0	0	0	1	0	0	0	0	0	0	0	0	0
ENSG000	FBXO33	MIMAT0000256	hsa-miR-181a-5p	**6**	1	0	0	0	1	0	1	0	0	1	1	1	1	0	0	1
ENSG000	FBXO33	MIMAT0000257	hsa-miR-181b-5p	**6**	0	0	0	0	1	0	1	0	0	1	1	1	1	0	0	0
ENSG000	HMGB3	MIMAT0000256	hsa-miR-181a-5p	2	0	0	0	0	0	0	1	0	0	0	0	0	1	0	0	0
ENSG000	HMGB3	MIMAT0000256	hsa-miR-181a-5p	2	0	0	0	0	0	0	1	0	0	0	0	0	1	0	0	0
ENSG000	HMGB3	MIMAT0000257	hsa-miR-181b-5p	2	0	0	0	0	0	0	1	0	0	0	0	0	1	0	0	0
ENSG000	HMGB3	MIMAT0000257	hsa-miR-181b-5p	2	0	0	0	0	0	0	1	0	0	0	0	0	1	0	0	0
ENSG000	HMGN2	MIMAT0000256	hsa-miR-181a-5p	2	0	0	0	0	1	0	1	0	0	0	0	0	0	0	0	0
ENSG000	HMGN2	MIMAT0000257	hsa-miR-181b-5p	1	0	0	0	0	1	0	0	0	0	0	0	0	0	0	0	0
ENSG000	ISPD	MIMAT0000270	hsa-miR-181a-3p	1	0	0	0	0	0	0	0	1	0	0	0	0	0	0	0	0
ENSG000	ISPD	MIMAT0000256	hsa-miR-181a-5p	2	0	0	0	0	1	0	1	0	0	0	0	0	0	0	0	0
ENSG000	ISPD	MIMAT0000257	hsa-miR-181b-5p	2	0	0	0	0	1	0	1	0	0	0	0	0	0	0	0	0
ENSG000	KIAA2022	MIMAT0000256	hsa-miR-181a-5p	**5**	0	0	0	0	1	0	0	0	0	1	1	1	1	0	0	0
ENSG000	KIAA2022	MIMAT0000257	hsa-miR-181b-5p	**5**	0	0	0	0	1	0	0	0	0	1	1	1	1	0	0	0
ENSG000	LUC7L3	MIMAT0000256	hsa-miR-181a-5p	1	0	0	0	0	1	0	0	0	0	0	0	0	0	0	0	0
ENSG000	LUC7L3	MIMAT0022692	hsa-miR-181b-3p	1	0	0	0	0	0	0	0	0	0	0	0	0	1	0	0	0
ENSG000	LUC7L3	MIMAT0000257	hsa-miR-181b-5p	1	0	0	0	0	1	0	0	0	0	0	0	0	0	0	0	0
ENSG000	MCM9	MIMAT0000256	hsa-miR-181a-5p	2	0	0	0	0	1	0	1	0	0	0	0	0	0	0	0	0
ENSG000	MCM9	MIMAT0000257	hsa-miR-181b-5p	2	0	0	0	0	1	0	1	0	0	0	0	0	0	0	0	0
ENSG000	MEAF6	MIMAT0000256	hsa-miR-181a-5p	**3**	0	0	0	0	1	0	0	0	0	0	1	0	1	0	0	0
ENSG000	MEAF6	MIMAT0022692	hsa-miR-181b-3p	1	0	0	0	0	0	0	0	0	0	0	0	0	1	0	0	0
ENSG000	MEAF6	MIMAT0000257	hsa-miR-181b-5p	**3**	0	0	0	0	1	0	0	0	0	0	1	0	1	0	0	0
ENSG000	MED8	MIMAT0000256	hsa-miR-181a-5p	**4**	0	0	0	0	1	0	0	0	0	0	1	1	1	0	0	0
ENSG000	MED8	MIMAT0000257	hsa-miR-181b-5p	**4**	0	0	0	0	1	0	0	0	0	0	1	1	1	0	0	0
ENSG000	METTL16	MIMAT0000270	hsa-miR-181a-3p	1	0	0	0	0	0	0	0	0	0	0	0	0	1	0	0	0
ENSG000	MFSD6	MIMAT0000256	hsa-miR-181a-5p	**3**	0	0	0	0	1	0	0	0	0	0	1	0	1	0	0	0
ENSG000	MFSD6	MIMAT0000257	hsa-miR-181b-5p	**3**	0	0	0	0	1	0	0	0	0	0	1	0	1	0	0	0
ENSG000	PLEKHJ1	MIMAT0000256	hsa-miR-181a-5p	**3**	0	0	0	0	1	0	0	0	0	0	1	0	1	0	0	0
ENSG000	PLEKHJ1	MIMAT0000257	hsa-miR-181b-5p	**3**	0	0	0	0	1	0	0	0	0	0	1	0	1	0	0	0
ENSG000	PROSC	MIMAT0000256	hsa-miR-181a-5p	0	1	0	0	0	0	0	0	0	0	0	0	0	0	0	0	1
ENSG000	PROSC	MIMAT0022692	hsa-miR-181b-3p	1	0	0	0	0	0	0	0	0	0	0	0	0	1	0	0	0
ENSG000	PRUNE	MIMAT0000270	hsa-miR-181a-3p	2	0	0	0	0	0	1	0	0	0	0	0	0	1	0	0	0
ENSG000	PRUNE	MIMAT0000256	hsa-miR-181a-5p	1	0	0	0	0	1	0	0	0	0	0	0	0	0	0	0	0
ENSG000	RBBP7	MIMAT0000256	hsa-miR-181a-5p	**5**	0	0	0	0	1	0	1	0	0	0	1	1	1	0	0	0
ENSG000	RBBP7	MIMAT0000257	hsa-miR-181b-5p	**5**	0	0	0	0	1	0	1	0	0	0	1	1	1	0	0	0
ENSG000	SCG2	MIMAT0000257	hsa-miR-181b-5p	1	0	0	0	0	1	0	0	0	0	0	0	0	0	0	0	0
ENSG000	SCOC	MIMAT0000256	hsa-miR-181a-5p	**4**	0	0	0	0	1	0	0	0	0	0	1	1	1	0	0	0
ENSG000	SCOC	MIMAT0000257	hsa-miR-181b-5p	**4**	0	0	0	0	1	0	0	0	0	0	1	1	1	0	0	0
ENSG000	SMYD4	MIMAT0022692	hsa-miR-181b-3p	1	0	0	0	0	0	0	0	0	0	0	0	0	1	0	0	0
ENSG000	SMYD4	MIMAT0000257	hsa-miR-181b-5p	1	0	0	0	0	1	0	0	0	0	0	0	0	0	0	0	0
ENSG000	ST7	MIMAT0000256	hsa-miR-181a-5p	1	0	0	0	0	0	0	1	0	0	0	0	0	0	0	0	0
ENSG000	ST7	MIMAT0000257	hsa-miR-181b-5p	1	0	0	0	0	0	0	1	0	0	0	0	0	0	0	0	0
ENSG000	TLE4	MIMAT0000256	hsa-miR-181a-5p	1	0	0	0	0	1	0	0	0	0	0	0	0	0	0	0	0
ENSG000	TLE4	MIMAT0000257	hsa-miR-181b-5p	1	0	0	0	0	1	0	0	0	0	0	0	0	0	0	0	0
ENSG000	TRIM37	MIMAT0000270	hsa-miR-181a-3p	1	0	0	0	0	0	0	0	0	0	0	0	0	1	0	0	0
ENSG000	ZDHHC15	MIMAT0000256	hsa-miR-181a-5p	2	0	0	0	0	1	0	1	0	0	0	0	0	0	0	0	0
ENSG000	ZDHHC15	MIMAT0022692	hsa-miR-181b-3p	1	0	0	0	0	0	0	0	0	0	0	0	0	1	0	0	0
ENSG000	ZDHHC15	MIMAT0000257	hsa-miR-181b-5p	1	0	0	0	0	0	0	1	0	0	0	0	0	0	0	0	0
ENSG000	ZNF367	MIMAT0000256	hsa-miR-181a-5p	2	0	0	0	0	1	0	1	0	0	0	0	0	0	0	0	0
ENSG000	ZNF367	MIMAT0000257	hsa-miR-181b-5p	1	0	0	0	0	0	0	1	0	0	0	0	0	0	0	0	0

Suppl. Table 3 (related to Figure 10). Human genes predicted to contain a seed sequence for *mir181ab1*.

SUPPLEMENTARY METHODS

Mouse work. Genotyping was done on DNA extracted from tail clipping. Mice were intratracheally administered a dose of $5x10^6$ pfu of adCre and lungs harvested 20 weeks after infection. For tumor maintenance experiments, mice were administered tamoxifen (2 mg/100 µl) at the age of 8 weeks on 3 consecutive days, and lungs harvested 8 weeks later. Blinding analyses were carried out when tumor burden, tumor number or tumor size was scored in the genetic experiment. Score of immunohistochemistry analyses was also blinded. For tumor formation assays, 5 x 10^4 *Kras*^{LA2}; *Rosa26Cre*^{ERT2}; *Mir181*$^{flox/flox}$ mouse lung cancer cells (KLA), previously treated for 2 days with adCre or adEmpty, were suspended in serum free DMEM and injected subcutaneously into the lower flanks of Rag2$^{-/-}$ mice (Charles River).

For pancreatic cancer initiation studies Ptf1a$^{Cre/+}$; Kras$^{LSL-G12D/+}$ and Ptf1a$^{Cre/+}$; *Kras*$^{LSL-G12D/+}$; *Mir181ab1*$^{flox/flox}$ mice were analyzed at 6 month of age. For survival studies, *Ptf1a*$^{Cre/+}$; *Kras*$^{LSL-G12D/+}$; *Trp53*$^{flox/flox}$ and *Ptf1a*$^{Cre/+}$; *Kras*$^{LSL-G12D/+}$; *Trp53*$^{flox/flox}$; *Mir181ab1*$^{flox/flox}$ mice were used and followed for signs of disease progression. Histopathological analysis was based on the classification consensus (73). Two stage tumorigenesis experiments were performed using *Kras*$^{FSF-G12D/+}$; *Rosa26*$^{FSF-CreERT2}$ (74), *Trp53*$^{FRT/FRT}$ and *Mir181ab1*$^{flox/flox}$ mutant mice. Tumors were induced by replication-deficient adenoviruses expressing Cre-recombinase (ad-Cre) delivered by intrapancreatic duct injection, as previously described (45, 75). Briefly, 8 to 10-week old Kras *Kras*$^{FSF-G12D/+}$; *Trp53*$^{FRT/FRT}$; *Rosa26*$^{FSF-CreERT2}$; *Mir181ab1*$^{flox/flox}$ were injected with 100 µl of 5 x 10^6 PFU Ad-Cre solution (Baylor College of Medicine, Viral Vector Production Core) into the common bile duct adjacent to the duodenum. Next, tumors were resected and isolated cancer cells (0.5 x 10^6) were mixed with matrigel (1:1) and transplanted to the flanks of NSG mice (NOD.SCID-IL2Rg-/-) were utilized for PanIN transplantation studies. Tumor volume was calculated every three days. Once the tumors reached 150 mm^3 mice were subjected to three (administered every other day) intraperitoneally injections of tamoxifen (1 mg/mouse) or vehicle control (corn oil) to induce *Mir181ab1* gene deletion.

Tumor dimensions were measured twice a week and tumor volume was calculated using Volume= $\pi/6$ x (length) x (width)^2.

Cell cycle assay. Cell cycle analysis was carried out with Click-iT® EdU Flow Cytometry Assay Kit (Invitrogen). Cells were seeded, treated with adCre for 48 h and cultured for additional 72 or 96 h with fresh medium. At the indicated time points, cells were treated following manufacturer's instructions. Cells were assessed with

FACSCanto II cytometer (BD Biosciences). Data were analyzed using FlowJo® software v9.3.

Pharmacological screen. KLA wt/wt and mut/mut cells pretreated with adCre for 48 h and seeded in 96-well plates. After overnight culture, different doses (previously established in pilot studies) of trametinib (MEKi), AZ08055 (mTORi), GSK2126458 (PI3Ki), alisertib (AURKAi), barasertib (AURKBi), pacritinib (JAK2/FLT3i), NVP-BEZ235 (Pi3K/mTORi), BI-2536 (PLK1i), neratinib (EGFR/HER2i), JQ1 (BRDi), CP673451 (PDGFRi), FGFRi, palbociclib (CDK4/6i), fredatinib (JAK2i), apicidin (HDACi), panobinostat (HDACi), IKK16 (IKB/IKKi), SP600125 (JNKi), BIX02189 (MEK5i) or dasatinib (BCR-ABL/SRC/c-KITi). All inhibitors were purchased from Selleck.

Pharmacological inhibitor treatment. KLA and KPC cells were treated with trametinib (MEKi, 10 µM), BIX02189 (MEK5i, 10 µM), SP600125 (JNKi, 20 µM), GSK2126458 (Pi3Ki, 0.1 µM), SB203580 (p38i, 20 µM), IKK-16 (IKKi, 1 µM), and LY294002 (AKTi, 10 µM) for 3 and 12 h. RNA was extracted at indicated time points and miR181a and miR181b expression was assessed by qPCR.

Western blot. Cells were seeded in 60 mm dishes, scrapped and lysed using a lysis buffer containing 1% NP-40, 150 mM NaCl, 50 mM Tris pH 7.4, 1 mM EDTA, 1% glycerol, supplemented with protease inhibitor cocktail (Roche), 0.5 M sodium fluoride and 200 mM sodium orthovanadate. 20 µg of protein samples were resolved by SDS PAGE and transferred to a nitrocellulose membrane (Bio-Rad). Membranes were blocked with 5% milk PBS-T for 1 h at room temperature. Primary antibodies were incubated overnight at 4º C and secondary antibodies were incubated for 1 h at room temperature. SuperSignal™ West Pico PLUS Chemiluminescent Substrate (34580; Thermo Scientific) or Lumigen ECL Ultra (TMA-100; Lumigen) and the Gel Imaging System (Bio-Rad) were used for protein detection and bands were quantified using ImageJ software.

RNA sequencing analysis. Raw fastq files were trimmed with Trimmomatic/0.36 and reads were aligned to the mm10 reference genome with STAR/2.5.1b aligner. Gene level counts were determined with STAR –*quantMode* option using gene annotations from GENCODE (vM13). QC assessments such as unique alignment counts, unique/multiple ratio or exon/intron ratio was derived with ngsutilsj-0.3-2180ca6 using the *bam-stats* option. Differential gene expression and all other pathway analysis are conducted with R/3.4.3. Samples were imported, normalized with trimmed mean of M-values (TMM) from the EdgeR/3.20.9 package and further transformed with VOOM from the Limma/3.34.9 package, resulting in a log2 normalized count matrix. A linear

model using the Limma/3.34.9 package was then used to obtain p-values, adjusted p-values and log-fold changes (LogFC).

Prediction of potential miR181ab1 targets. Genes downregulated upon exogenous miR181ab1 expression were analyzed based on target prediction databases. The following databases were used for target prediction: TargetScan release 5.1 (3), PicTar (4), PITA release 6 (5), miranda release sept2008 (6), microcosm release 5 (7) and IoRNA database (8). All these information and validated miRNA targets were used with the methods developed in (8).

SPECIFIC MATERIAL INFORMATION

REAGENT	SOURCE	IDENTIFIER
Antibodies		
MUC5AC	Thermo Fisher Scientific	Cat# 145P1
Ki67	BD Bioscience	Cat# 550609
Cleaved Caspase 3	Cell Signaling Technologies	Cat# 9664
anti-Mouse IgG Biotinylated	Cell Signaling Technologies	Cat# 14709
anti-Rabbit IgG Biotinylated	Cell Signaling Technologies	Cat# 14708
Phospho-H3	Cell Signaling Technologies	Cat# 9701
KRAS	Calbiochem	Cat# OP24-100M6
RASSF1A	eBioscience	Cat# 14-6888-82
β-TUBULIN	Sigma	Cat# T4026
HSP90	Santa Cruz Biotechnologies	Cat# SC-69703
Bacterial and Virus Strains		
Adenovirus-Cre	Baylor College of Medicine, Viral Vector Production Core	Cat# Ad-Cre
Adenovirus-Cre	Iowa University	Cat# Ad5-CMV Cre
Adenovirus-Empty	Iowa University	Cat# Ad5-CMV Empty
Plasmids		
pMWX mir181wt/wt, wt/mut, mut/wt, mut/mut	(9)	
pX333	A gift from Andrea Ventura	Addgene plasmid # 64073 ;
pMAX GFP	Lonza	
pLenti6-CMV KRASG12D	This study	Mutant KRAS from Ras initiative (Addgene)
psiCHECK™-2	Promega	
pSIN-EF2-Nexmif-Puro	This study	
Chemicals, Peptides, and Recombinant Proteins		
Hydrogen Peroxide	Thermo Fisher Scientific	Cat# H325-500
Tamoxifen	Sigma-Aldrich	Cat# T5648

2-hydroxypropyl-β-cyclodextrin	Sigma-Aldrich	Cat# H107
Bovine Serum Albumin (BSA)	Thermo Fisher Scientific	Cat# BP9703100
TRIzol	Invitrogen	Cat#15596026
SYBRGreen	Applied Biosystems	Cat#4309155

Critical Commercial Assays

DAB Substrate Kit	Abcam	Cat# ab64238
Vectastain ABC kit	Vector Laboratories	Cat# PK-6100
CellTiter 96 ® Aqueous Non-Radiactive Cell Proliferation Assay, MTS	Promega	Cat#G5421
CellTiterGLO	Promega	Cat#G7570
DyNAmo cDNA synthesis kit	F470, New England Biolas	Cat#E6560S
TaqMan MicroRNA Assay miR181a	Thermo Fisher Scientific	Cat#4427975 ID000480
TaqMan MicroRNA Assay miR181b	Thermo Fisher Scientific	Cat#4427975 ID001098
Click-iT® EdU Flow Cytometry Assay Kit	Invitrogen	Cat#C10634
Dual Luciferase Report Assay System	Promega	Cat#E1910
miRNA Northern Blot Assay Kit including 2 gels	Signosis	NB-0001

Primers

mir181ab1 Genotyping	Sigma-Aldrich	1: taacttgagaaaacctaagtgg<br>2: gggagcttagttacaggctg<br>3: tccctggaatcaagtctac
sgRNA CRISPR/Cas9 miR181	Sigma-Aldrich	5'#1: cagtgaacattcaacgctgt<br>3'#2: tgtcggtgggttgaactgtg<br>3'#4: ctgaacaatgaatgcaactg
miR181 KO CRISPR/Cas9 system	Sigma-Aldrich	Fw: tccatctctggaactagcccaa<br>Rev: ccagaagatgctgacgaattgga
mmu C77370	Sigma-Aldrich	Fw: ggataaccaacaagataaagttattgc<br>Rev: acttcctggtcctctgagtcat
mmu Meaf6	Sigma-Aldrich	Fw: ctggccgagctggttaag<br>Rev: tctctccaagtttgcaagtgttt
mmu Dek	Sigma-Aldrich	Fw: aaagccttctggcaaaccat<br>Rev: ccagaactgttccgttccttt
mmu Fbxo33	Sigma-Aldrich	Fw: gcatctacttggagctggtgt<br>Rev: tccaaaaagactaaacttctggagat
mmu Mfsd6	Sigma-Aldrich	Fw: gccgtgcttctgagatacaa<br>Rev: taggtgaggcggagtgagtt
mmu Plekhj1	Sigma-Aldrich	Fw: agtggcccaggaagaacc<br>Rev: gctcttggctacagcattca
mmu Med8	Sigma-Aldrich	Fw: atgccaggtgctccaaac<br>Rev: tttattccacttggcattttcc
mmu Scoc	Sigma-Aldrich	Fw: tgaggcttcctacccatcc

		Rev: cctcgaacgccaactcttta
mmu Rbbp7	Sigma-Aldrich	Fw: acgcaagatggcgagtaaag<br>Rev: cagattttatactcttcgttgatgaca
mmu Dtx4	Sigma-Aldrich	Fw: ccggactgcaagaccatc<br>Rev: tgaagctcttcccaggattc
mmu Cebpα	Sigma-Aldrich	Fw: caagaacagcaacgagtaccg<br>Rev: gtcactggtcaactccagcac
mmu Cebpβ	Sigma-Aldrich	Fw: atcgacttcagcccctacct<br>Rev: tagtcgtcggcgaagagg
mmu cMyb	Sigma-Aldrich	Fw: agaccccgacacagcatcta<br>Rev: cagcagcccatcgtagtcat
mmu Evi1	Sigma-Aldrich	Fw: aagtaatgagtgtgcctatggc<br>Rev: agttgactctcgaagctcaaac
mmu Meis1	Sigma-Aldrich	Fw: gcaaagtatgccaggggagta<br>Rev: tcctgtgttaagaaccgaggg
mmu Gata2	Sigma-Aldrich	Fw: caccccgccgtattgaatg<br>Rev: cctgcgagtcgagatggttg
mmu Gata3	Sigma-Aldrich	Fw: aagctcagtatccgctgacg<br>Rev: gtttccgtagtaggacgggac
Mmu Foxa2	Sigma-Aldrich	Fw: tccgactggagcagctactac<br>Rev: gcgcccacataggatgaca
Hm GATA3	Sigma-Aldrich	Fw: GCCCCTCATTAAGCCCAAG<br>Rev: TTGTGGTGGTCTGACAGTTCG

Experimental Models: Cell Lines		
KPC (mouse PDAC cell lines)	In this study	N/A
KPCmiR181$^{-/-}$ (mouse PDAC cell lines)	In this study	N/A
KLA (mouse LUAD cell line)	In this study	N/A
MEF	In this study	N/A
H6c7	Kerafast	
3KT	(10)	
NCI-H2126	ATCC	Cat# CCL-256
NCI-H1792	ATCC	Cat# CRL-5895

Experimental Models: Organisms/Strains		
Mouse: *Kras*$^{LSL\text{-}G12D}$	The Jackson Laboratories	Strain# 008179
Mouse: *p53*$^{lox/lox}$	The Jackson Laboratories	Strain# 008462
Mouse: *Ptf1a*^{Cre}	(Kawaguchi et al., 2002)	MGI# 2387812
Mouse: *mir181*$^{-/-}$	(9)	N/A
Mouse: *mir181*$^{lox/lox}$	(9)	N/A
Mouse: *ROSA26*$^{FSF\text{-}CreERT2}$	(11)	N/A
Mouse: *Kras*$^{FSF\text{-}G12D}$	(11)	N/A
Mouse: *Trp53*$^{FRT/FRT}$	(12)	MGI:5641750
Mouse: *NOD.SCID-*	The Jackson Laboratories	Strain# 005557

IL2Rg$^{-/-}$ (NSG)		

Software, Algorithms and Data bases		
Prism 7	GraphPad	https://www.graphpad.com/; RRID:SCR_002798
Excel for Mac 2016	Microsoft	https://www.microsoft.com/en-us/; RRID:SCR_016137
Leica Application Suite X (LAS X)	Leica	https://www.leica-microsystems.com/ RRID:SCR_013673
ImageJ – Fiji package	Freeware	http://fiji.sc; RRID:SCR_002285
Flo-Jo	V9.3	
Ingenuity Pathway Analysis		http://www.ingenuity.com
SPSS	V13.0	
Horos	GNU Lesser General Public License, Version 3.0	https://www.horosproject.org/
LIMMA (Linear Models for Microarray Data)	(1)	
R/bioconductor	(2)	
TargetScan release 5.1	(3)	
PicTar	(4)	
PITA release 6	(5)	
miranda release sept2008	(6)	
microcosm release 5	(7)	
loRNA database	(8)	
The Cancer Genome Atlas (TCGA)		https://tcga-data.nci.nih.gov/publications/tcga
International Cancer Genome consortium (ICGC)	(13)	

Other		
RPMI 1640 Medium	Corning	Cat# MT10017CV
DMEM Medium	Corning	Cat# MT10040CV
Keratinocyte-SFM Medium	Thermo Fisher Scientific	Cat# 17005042
Fetal bovine serum	Thermo Fisher Scientific	Cat# 10500056
PBS	Corning	Cat# MT21031CV
Trypsin-EDTA 0.25%	Corning	Cat# MT25053CI
Bovine Pituitary Extract (BPE)	Thermo Fisher Scientific	Cat# 13028014
RBC Lysis Buffer	Thermo Fisher Scientific	Cat# 00-4333-57
Matrigel	Corning	Cat# 354234
miR-181a-5p(HS)	Signosis	HP-0081
miR-181b-5p(HS)	Signosis	HP-0082

CONTACT FOR REAGENT AND RESOURCE SHARING

Further information and requests for resources and reagents should be directed to and will be fulfilled by the corresponding authors, Alejandro Sweet-Cordero (Alejandro.Sweet-Cordero@ucsf.edu) or Silvestre Vicent Cambra (silvevicent@unav.es).

SUPPLEMENTARY REFERENCES

1. Smyth GK. Linear models and empirical bayes methods for assessing differential expression in microarray experiments. *Stat Appl Genet Mol Biol.* 2004;3(Article3.
2. Gentleman RC, Carey VJ, Bates DM, Bolstad B, Dettling M, Dudoit S, Ellis B, Gautier L, Ge Y, Gentry J, et al. Bioconductor: open software development for computational biology and bioinformatics. *Genome Biol.* 2004;5(10):R80.
3. Friedman RC, Farh KK, Burge CB, and Bartel DP. Most mammalian mRNAs are conserved targets of microRNAs. *Genome Res.* 2009;19(1):92-105.
4. Lall S, Grun D, Krek A, Chen K, Wang Y-L, Dewey CN, Sood P, Colombo T, Bray N, Macmenamin P, et al. A genome-wide map of conserved microRNA targets in C. elegans. *Current biology : CB.* 2006;16(5):460-71.
5. Kertesz M, Iovino N, Unnerstall U, Gaul U, and Segal E. The role of site accessibility in microRNA target recognition. *Nature genetics.* 2007;39(10):1278-84.
6. Betel D, Koppal A, Agius P, Sander C, and Leslie C. Comprehensive modeling of microRNA targets predicts functional non-conserved and non-canonical sites. *Genome biology.* 2010;11(8):R90.
7. Griffiths-Jones S, Saini HK, van Dongen S, and Enright AJ. miRBase: tools for microRNA genomics. *Nucleic acids research.* 2008;36(Database issue):D154-8.
8. Guruceaga E, and Segura V. Functional interpretation of microRNA-mRNA association in biological systems using R. *Comput Biol Med.* 2014;44(124-31.
9. Fragoso R, Mao T, Wang S, Schaffert S, Gong X, Yue S, Luong R, Min H, Yashiro-Ohtani Y, Davis M, et al. Modulating the strength and threshold of NOTCH oncogenic signals by mir-181a-1/b-1. *PLoS Genet.* 2012;8(8):e1002855.
10. Sato M, Vaughan MB, Girard L, Peyton M, Lee W, Shames DS, Ramirez RD, Sunaga N, Gazdar AF, Shay JW, et al. Multiple oncogenic changes (K-RAS(V12), p53 knockdown, mutant EGFRs, p16 bypass, telomerase) are not sufficient to confer a full malignant phenotype on human bronchial epithelial cells. *Cancer Res.* 2006;66(4):2116-28.
11. Schonhuber N, Seidler B, Schuck K, Veltkamp C, Schachtler C, Zukowska M, Eser S, Feyerabend TB, Paul MC, Eser P, et al. A next-generation dual-recombinase system for time- and host-specific targeting of pancreatic cancer. *Nature Medicine.* 2014;20(11):1340-7.
12. Lee CL, Moding EJ, Huang XF, Li YF, Woodlief LZ, Rodrigues RC, Ma Y, and Kirsch DG. Generation of primary tumors with Flp recombinase in FRT-flanked p53 mice. *Dis Model Mech.* 2012;5(3):397-402.

13. Bailey P, Chang DK, Nones K, Johns AL, Patch AM, Gingras MC, Miller DK, Christ AN, Bruxner TJC, Quinn MC, et al. Genomic analyses identify molecular subtypes of pancreatic cancer. *Nature.* 2016;531(7592):47-+.
